# β‐Adducin Restrains PLA2G4A‐Dependent Lysosomal Membrane Permeabilization and Neuronal Death in Ischemic Stroke

**DOI:** 10.1002/advs.78183

**Published:** 2026-10-06

**Authors:** Hui‐qin Li, Pin‐yi Liu, Dan Wu, Fei‐yu Ma, Yi Bai, Sheng‐nan Xia, Hai‐yan Yang, Xin‐yu Bao, Yun Xu, Xiang Cao

**Affiliations:** ^1^ Department of Neurology Nanjing Drum Tower Hospital Affiliated Hospital of Medical School Nanjing University Nanjing China; ^2^ State Key Laboratory of Pharmaceutical Biotechnology and Institute of Translational Medicine For Brain Critical Diseases Nanjing University Nanjing China; ^3^ Nanjing Neurology Clinical Medical Centre and Centre for Central Nervous Disease and Neuroscience Nanjing China; ^4^ Department of Neurology College of Life Science Joint Institute of Nanjing Drum Tower Hospital for Life and Health Nanjing Normal University Nanjing China; ^5^ Jiangsu Key Laboratory For Molecular Medicine Medical School of Nanjing University Nanjing China

**Keywords:** β‐adducin, ischemic stroke, lysosomal membrane permeabilization, m^6^A modification, PLA2G4A

## Abstract

Failure to preserve vulnerable neurons in the ischemic penumbra remains a barrier in ischemic stroke. Lysosomal membrane permeabilization (LMP) is increasingly recognized as a critical event in ischemic neuronal death, yet the endogenous mechanisms that preserve lysosomal integrity remain poorly defined. Here, we identify β‐adducin (*Add2*) as an endogenous protector of lysosomal homeostasis in ischemic neurons. We found that β‐adducin was markedly reduced in neurons after cerebral ischemia in both middle cerebral artery occlusion and oxygen‐glucose deprivation/reoxygenation models. Neuron‐specific knockdown of *Add2* increased infarct volume, worsened neurological deficits, and enhanced neuronal apoptosis. Mechanistically, β‐adducin interacted with cytosolic phospholipase A2 group IVA (PLA2G4A) and restricted its accumulation on lysosomes. Loss of β‐adducin enhanced lysosomal localization of PLA2G4A, promoted LMP, increased cytosolic leakage of cathepsins, and exacerbated neuronal injury, whereas silencing PLA2G4A attenuated these effects. Upstream, ischemic stress reduced FTO expression, increased *N* ^6^‐methyladenosine (m^6^A) modification of *Add2* mRNA, and accelerated *Add2* transcript decay. Conversely, FTO overexpression restored β‐adducin expression and alleviated ischemic neuronal injury. Together, these findings identify β‐adducin as a key component of an endogenous lysosomal defense pathway in ischemic stroke and suggest that reduced FTO‐mediated demethylation and consequent β‐adducin loss contribute to PLA2G4A‐associated lysosomal injury and neuronal death.

## Graphical abstract

This study identifies β‐adducin as a regulator of lysosomal integrity in ischemic neurons. Ischemia‐associated FTO loss increases m^6^A modification and destabilizes *Add2* mRNA, reducing β‐adducin expression and promoting PLA2G4A accumulation at lysosomes. The resulting lysosomal membrane damage and cathepsin release contribute to neuronal injury, revealing a potential downstream target for ischemic stroke.

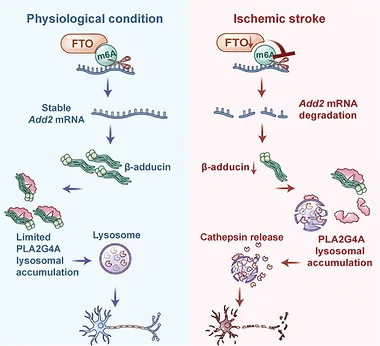

## 1 Introduction

Ischemic stroke remains a leading cause of mortality and long‐term disability worldwide [1]. The primary goal of acute stroke treatment is to restore cerebral blood flow as quickly as possible to salvage vulnerable neurons in the ischemic penumbra. Intravenous thrombolysis and mechanical thrombectomy are currently the main reperfusion therapies for acute ischemic stroke [2, 3]. However, their clinical benefit is limited by a narrow therapeutic time window, strict eligibility criteria, and the risk of ischemia‐reperfusion injury. Consequently, many patients still develop progressive neuronal loss and persistent neurological deficits despite successful recanalization [4, 5, 6, 7]. These limitations highlight the need to define endogenous mechanisms that preserve neuronal viability during ischemic stress and to develop effective neuroprotective strategies.

Among the many pathological events triggered by cerebral ischemia, lysosomal integrity has emerged as a critical determinant of neuronal survival [8, 9, 10]. Lysosomes are essential for intracellular degradation and metabolic homeostasis, but they can also become a source of potent hydrolases once membrane integrity is compromised. Severe ischemic injury can induce lysosomal membrane permeabilization (LMP), resulting in the release of lysosomal enzymes into the cytosol and ultimately causing irreversible neuronal injury [11, 12, 13, 14]. Increasing evidence suggests that cytosolic phospholipase A2 group IVA (PLA2G4A) may contribute to lysosomal membrane injury. Upon membrane translocation, PLA2G4A hydrolyzes membrane phospholipids and promotes lysosomal membrane damage [15, 16, 17]. However, the endogenous mechanisms that restrain PLA2G4A and preserve lysosomal stability in neurons under ischemic conditions remain poorly understood.

β‐adducin, encoded by the gene *Add2*, is a membrane cytoskeleton‐associated protein that participates in spectrin‐actin network assembly and stabilizes membrane‐associated protein complexes [18, 19, 20]. In the nervous system, β‐adducin has been implicated in synaptic plasticity, long‐term memory formation, and cognitive function [21, 22, 23]. Given its established role in membrane cytoskeletal organization, β‐adducin is a plausible candidate for maintaining subcellular membrane stability under pathological stress. Nevertheless, whether β‐adducin contributes to lysosomal protection during ischemic injury has not been explored. In particular, it remains unknown whether β‐adducin is altered in ischemic neurons and, if so, whether such changes are functionally linked to lysosomal injury and neuronal death.

An additional unresolved question is how β‐adducin expression is regulated during ischemic stress. Brain‐specific *Add2* transcripts contain unusually long 3' untranslated regions (3' UTRs), suggesting potential susceptibility to post‐transcriptional regulation [24, 25]. *N* ^6^‐methyladenosine (m^6^A), the most abundant internal mRNA modification in the mammalian brain, is markedly altered after stroke, and the demethylase FTO has emerged as an important regulator of ischemic brain injury [26, 27, 28, 29]. In addition, publicly available methylated RNA immunoprecipitation sequencing datasets indicate that the *Add2* mRNA carries m^6^A modification [28]. These observations raise the possibility that ischemic stress may reduce β‐adducin expression through FTO‐dependent epitranscriptomic dysregulation, thereby increasing neuronal vulnerability to lysosomal injury.

Here, we identify β‐adducin as an endogenous protector of lysosomal integrity in ischemic neurons. We further examine whether ischemia‐associated m^6^A dysregulation contributes to the loss of β‐adducin and thereby promotes PLA2G4A‐associated lysosomal injury. By addressing these questions, our study defines a β‐adducin‐centered lysosomal defense pathway in ischemic stroke and provides a mechanistic framework linking post‐transcriptional regulation to lysosomal vulnerability and neuronal death.

## 2 Results

### 2.1 β‐Adducin is Downregulated After Ischemic Stroke and is Predominantly Enriched in Neurons

To determine whether β‐adducin is altered after ischemic injury, we first examined our previously generated proteomic dataset from ischemic penumbra following middle cerebral artery occlusion (MCAO) [30]. Among the adducin family members, β‐adducin, encoded by *Add2*, exhibited the greatest decrease relative to the sham group (Figure 1A). This reduction was subsequently validated by qPCR and western blot analyses, which showed that *Add2* mRNA and β‐adducin protein levels were markedly decreased in the ischemic cortex at 1, 3, and 7 days after MCAO. In parallel, *Add1* mRNA was also decreased, whereas α‐adducin protein showed no significant change and *Add3* mRNA, encoding γ‐adducin, remained unaltered (Figure 1B–E).

**FIGURE 1:**
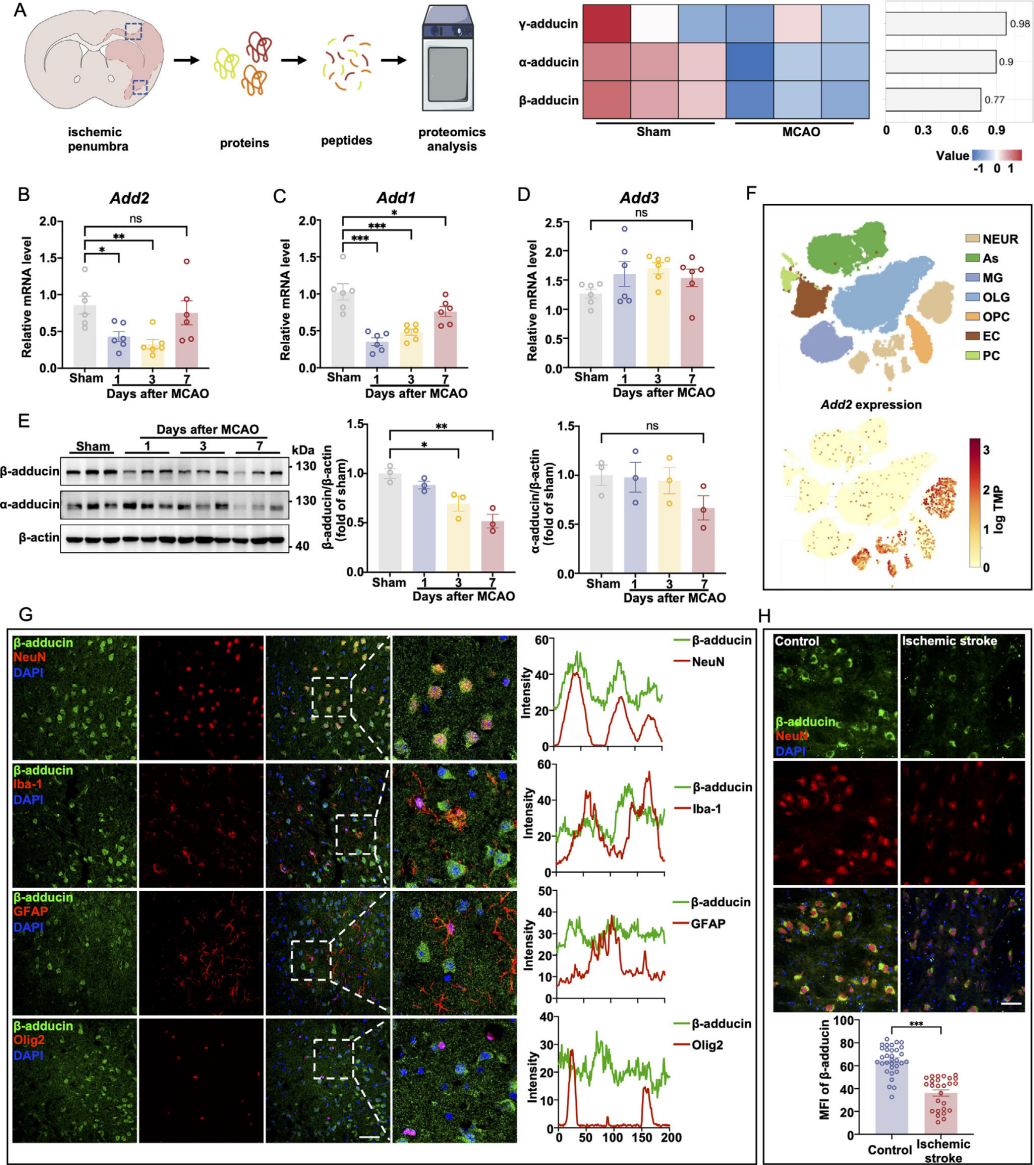
β‐adducin is downregulated after ischemic injury and is predominantly enriched in neurons (A) Proteomic heatmap showing the abundance of adducin family members (α‐, β‐, and γ‐adducin) in brain tissues from sham mice and the ischemic penumbra of mice 3 days after MCAO. (B–D) Relative mRNA expression levels of *Add2* (B), *Add1* (C), and *Add3* (D), encoding β‐adducin, α‐adducin, and γ‐adducin respectively, in the ischemic penumbra at 1, 3, and 7 days after MCAO relative to sham‐operated animals (*n* = 6, one‐way ANOVA, *p < 0.05, **p < 0.01, ***p < 0.001, ns, not significant). (E) Representative western blots and quantification of β‐adducin and α‐adducin protein levels at 1, 3, and 7 days after MCAO. β‐actin was used as the internal loading control (*n* = 3, one‐way ANOVA, *p < 0.05, **p < 0.01, ns, not significant). (F) UMAP plots of single‐cell RNA sequencing (scRNA‐seq) data derived from Ref.  [31], showing the clustering of distinct neural cell populations and the enrichment of *Add2* transcripts in neurons. Abbreviations: NEUR, neurons; As, astrocytes; MG, microglia; OLG, oligodendrocytes; OPC, oligodendrocyte precursor cells; EC, endothelial cells; PC, pericytes. (G) Representative immunofluorescence images and spatial fluorescence intensity profiles illustrating the cellular localization of β‐adducin in the mouse brain. Sections were co‐stained with cell‐type‐specific markers including NeuN for neurons, Iba‐1 for microglia, GFAP for astrocytes, and Olig2 for oligodendrocytes. Nuclei were counterstained with DAPI. Scale bar: 50 µm. (H) Representative immunofluorescence images and quantification of β‐adducin mean fluorescence intensity (MFI) within NeuN‐positive neurons in peri‐infarct middle temporal gyrus tissues from ischemic stroke patients and region‐matched middle temporal gyrus tissues from non‐neurological controls (*n* = 3, unpaired *t*‐test, ***p < 0.001). Scale bar, 50 µm. Data are expressed as the mean ± SEM. The original blot can be found in Figure S15.

We next asked which cell types predominantly express β‐adducin in the central nervous system by analyzing a publicly available single‐cell RNA sequencing (scRNA‐seq) dataset of the adult mouse brain [31]. The transcriptomic profile demonstrated that *Add2* is predominantly enriched in neurons, whereas *Add1* is more broadly distributed across multiple neural cell populations (Figure 1F; Figure S1A). Immunofluorescence staining further demonstrated that β‐adducin was predominantly localized to NeuN‐positive neurons, with minimal overlap with Iba‐1‐positive microglia, GFAP‐positive astrocytes, or Olig2‐positive oligodendrocytes (Figure 1G). In addition, immunostaining showed that β‐adducin expression in the neurons was reduced at 1 and 3 days after MCAO (Figure S1B). We further assessed β‐adducin expression in postmortem brain tissues derived from ischemic stroke patients and non‐neurological controls. Immunofluorescence staining showed that β‐adducin was predominantly observed in neurons in human brain tissue, and its mean fluorescence intensity (MFI) was significantly decreased in peri‐infarct regions of the middle temporal gyrus from stroke patients compared with region‐matched non‐neurological controls (Figure 1H). Together, these data indicate that β‐adducin is predominantly expressed in neurons and is downregulated after ischemic injury in both mice and humans.

### 2.2 Neuron‐Specific Knockdown of β‐Adducin Aggravates Ischemic Brain Injury

To determine the in vivo role of neuronal β‐adducin in ischemic stroke, we selectively knocked down *Add2* in neurons by tail‐vein administration of AAV three weeks prior to MCAO (Figure 2A; Figure S2A). Immunofluorescence staining confirmed efficient AAV transduction in NeuN‐positive neurons (Figure 2B; Figure S2B), and the reduction in β‐adducin expression was further validated by qPCR and western blot analyses (Figure S2C,D).

**FIGURE 2:**
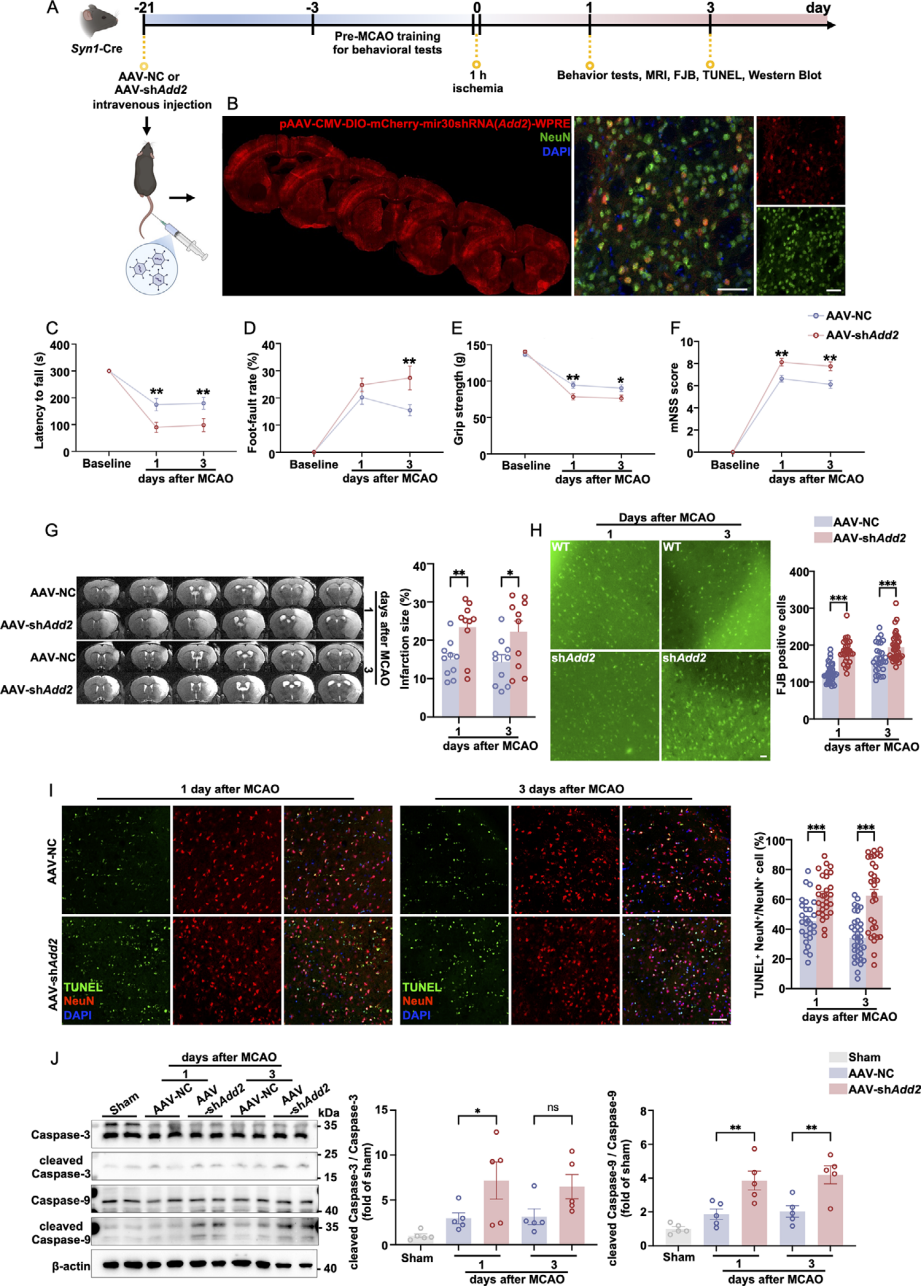
Neuron‐specific knockdown of β‐adducin aggravates ischemic brain injury. (A) Schematic diagram illustrating the experimental design and timeline. *Syn1*‐Cre mice received tail‐vein injection of AAV‐NC or AAV‐sh*Add2* prior to MCAO surgery. (B) Representative immunofluorescence images confirming the neuron‐specific expression of the Cre‐dependent AAV vector. Brain sections show AAV‐derived mCherry fluorescence and NeuN staining. Scale bar, 50 µm. (C–F) Evaluation of neurological function in AAV‐NC and AAV‐sh*Add2*‐treated mice according to the latency to fall in the rotarod test (C), foot‐fault rate (D), grip strength (E), and mNSS score (F) at baseline and at 1 and 3 days after MCAO (*n* = 19 for AAV‐NC, *n* = 16 for AAV‐sh*Add2*, two‐way ANOVA, *p < 0.05, **p < 0.01). (G) Representative T2‐weighted MRI scans and quantitative analysis of cerebral infarct volume at 1 and 3 days after MCAO (*n* = 10, two‐way ANOVA, *p < 0.05, **p < 0.01). (H) FJB staining and the quantification of FJB‐positive degenerating neurons in the ischemic penumbra (*n* = 4, two‐way ANOVA, ***p < 0.001). Scale bar, 20 µm. (I) Representative images of TUNEL/NeuN double staining in the ischemic penumbra at 1 and 3 days after MCAO. The percentage of TUNEL‐positive neurons among NeuN‐positive cells was quantified (*n* = 4, two‐way ANOVA, ***p < 0.001). Scale bar, 50 µm. (J) Representative western blots and quantification of the expression of apoptosis‐related proteins, including Caspase‐3, cleaved Caspase‐3, Caspase‐9, and cleaved Caspase‐9 (*n* = 5, one‐way ANOVA, *p < 0.05, **p < 0.01). β‐actin was used as the internal loading control. Data are expressed as the mean ± SEM. The original blot can be found in Figure S15.

Following neuron‐specific *Add2* knockdown, mice were subjected to MCAO and evaluated for neurological deficits at 1 and 3 days after surgery. Compared with AAV‐NC mice, mice with β‐adducin knockdown showed worse performance in the rotarod, foot‐fault, and grip strength tests, together with higher modified neurological severity scores (mNSS), indicating aggravated neurological dysfunction (Figure 2C–F). MRI analysis further showed a larger infarct volume in AAV‐sh*Add2* mice than in AAV‐NC mice at both time points (Figure 2G). Consistent with these findings, Fluoro‐Jade B (FJB) staining revealed more degenerating neurons in the ischemic penumbra after β‐adducin knockdown (Figure 2H). Moreover, TUNEL/NeuN double staining revealed a significantly higher proportion of TUNEL‐positive neurons in AAV‐sh*Add2* mice at both 1 and 3 days after MCAO (Figure 2I), further supporting enhanced ischemia‐induced apoptotic neuronal death. Western blot analysis of penumbral tissue showed increased levels of cleaved Caspase‐3 and cleaved Caspase‐9 in AAV‐sh*Add2* mice at 1 and 3 days after MCAO (Figure 2J). These results indicate that neuron‐specific β‐adducin deficiency aggravates MCAO‐induced neuronal apoptosis and ischemic brain injury.

### 2.3 β‐Adducin Protects Primary Neurons Against OGD/R‐Induced Injury

Given the complex microenvironment of the ischemic brain, we next turned to primary neurons to examine the role of β‐adducin under controlled in vitro ischemic conditions. We first assessed the expression pattern of β‐adducin after OGD/R and then examined its functional significance. qPCR analysis showed that *Add2* mRNA progressively decreased at 3, 12, and 24 h after OGD/R (Figure 3A). This reduction was further confirmed at the protein level by Western blot analysis and by decreased β‐adducin immunofluorescence intensity (Figure 3B,C). In contrast, α‐adducin exhibited no significant decline in primary neurons following OGD/R (Figure S3A). A similar expression pattern was observed in the HT‐22 cells after OGD/R (Figure S3B).

**FIGURE 3:**
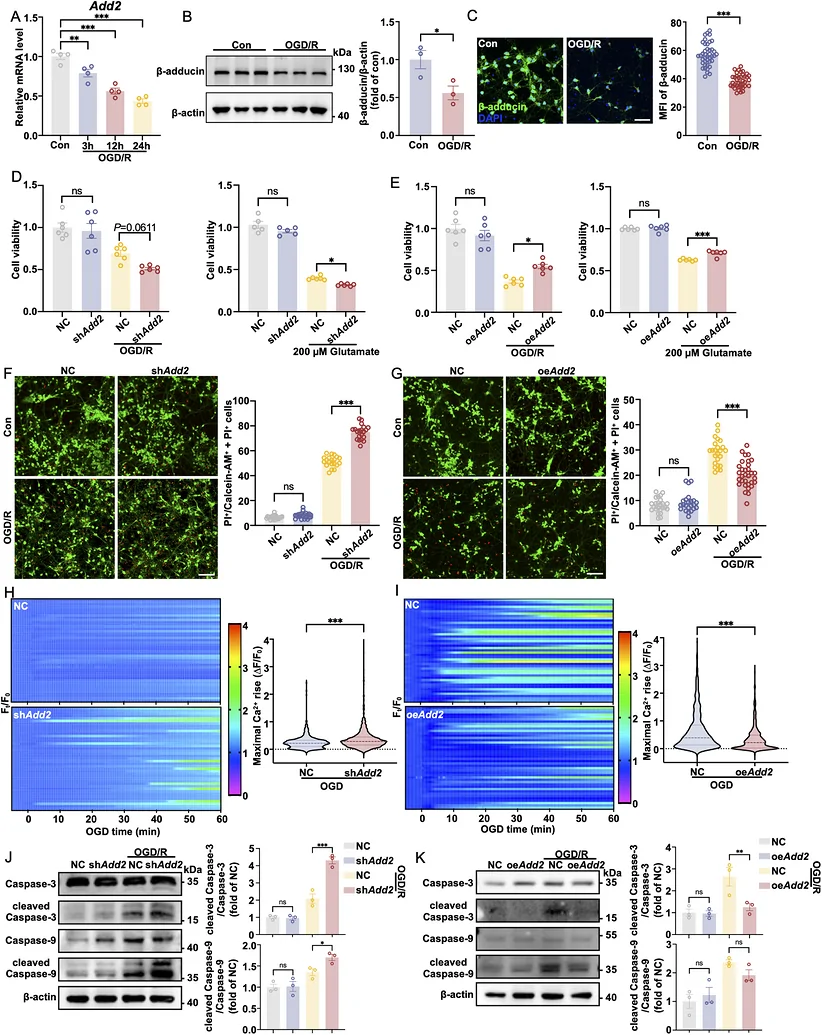
β‐adducin protects primary neurons against OGD/R‐induced injury. (A) Relative mRNA expression levels of *Add2* in primary neurons subjected to OGD/R for 3, 12, and 24 h (*n* = 4, one‐way ANOVA, **p < 0.01, ***p < 0.001). (B) Protein levels of β‐adducin in primary neurons after 12 h of OGD/R treatment (*n* = 3, unpaired *t*‐test, *p < 0.05). (C) The MFI of β‐adducin in primary neurons subjected to 12 h OGD/R (*n* = 3, unpaired *t*‐test, ***p < 0.001). Scale bar, 50 µm. (D,E) Cell viability assays assessing the effects of *Add2* knockdown (sh*Add2*) (D) and *Add2* overexpression (oe*Add2*) (E) in primary neurons exposed to either 12 h OGD/R or 200 µm glutamate toxicity (*n* = 5 to 6, one‐way ANOVA, *p < 0.05, ***p < 0.001, ns, not significant). NC indicates the corresponding negative control vectors. (F,G) Representative images and quantification of Live/Dead staining using Calcein‐AM (green, live cells) and PI (red, dead cells) in sh*Add2* (F) and oe*Add2* (G) neurons following 12 h of OGD/R insult (*n* = 3, one‐way ANOVA, ***p < 0.001). Scale bar, 100 µm. (H,I) Intracellular calcium dynamics of primary neurons during 60 min of OGD. Representative Ca^2+^ fluorescence intensity traces and the maximal Ca^2+^ rise of *Add2* knockdown (H) and overexpression (I) neurons (*n* = 3, unpaired *t*‐test, ***p < 0.001). (J,K) Protein expression of Caspase‐3, cleaved Caspase‐3, Caspase‐9, and cleaved Caspase‐9 relative to β‐actin in *Add2* knockdown (J) and overexpression (K) neurons following OGD/R insult (*n* = 3, one‐way ANOVA, **p < 0.01, ***p < 0.001, ns, not significant). Data are expressed as the mean ± SEM. The original blot can be found in Figure S15.

To evaluate its function, β‐adducin was efficiently knocked down or overexpressed in primary neurons (Figure S4A–D). Subsequent CCK‐8 assays showed that β‐adducin knockdown further reduced cell viability in primary neurons exposed to either OGD/R or glutamate stimulation, whereas β‐adducin overexpression significantly preserved neuronal viability in both models (Figure 3D,E). Consistent with these findings, LDH release assays showed that *Add2* knockdown increased OGD/R‐induced cytotoxicity, whereas β‐adducin overexpression attenuated LDH release following OGD/R (Figure S4E,F). These changes were also visualized by Calcein‐AM/PI dual staining, which showed increased neuronal death after *Add2* knockdown and reduced death after β‐adducin overexpression under OGD/R conditions (Figure 3F,G). Similar results were observed in HT‐22 cells (Figures S5A–D and s6 A–D).

Given that intracellular calcium overload triggers a lethal cascade in ischemic neurons [32], we performed live‐cell calcium imaging to assess calcium homeostasis. OGD exposure induced a marked increase in intracellular calcium levels in primary neurons. Notably, this abnormal calcium accumulation was further enhanced by β‐adducin knockdown but attenuated by β‐adducin overexpression (Figure 3H,I; Figure s4 G,H). Consistent with these changes, western blot analysis indicated that β‐adducin knockdown increased the cleavage of Caspase‐3 and Caspase‐9 after OGD/R, while β‐adducin overexpression suppressed the activation of these pro‐apoptotic cascades (Figure 3J,K). Comparable changes in calcium dynamics and apoptotic signaling were also observed in HT‐22 cells (Figures S5E,F and S6E,F). Collectively, these data indicate that β‐adducin protects neurons against ischemic‐like injury in vitro.

### 2.4 β‐Adducin Interacts With PLA2G4A and Limits Its Lysosomal Accumulation During OGD/R

To explore the mechanism underlying the protective effect of β‐adducin, we sought to identify β‐adducin‐interacting proteins by immunoprecipitation‐mass spectrometry (IP‐MS) in primary neurons overexpressing Flag‐tagged β‐adducin (Figure 4A). Based on peptide abundance, confidence score, and potential functional relevance, PLA2G4A, Dpp3, and IMPACT were selected as candidate β‐adducin‐interacting proteins (Figure 4B). To validate these interactions, HEK293T cells were co‐transfected with Flag‐*Add2* and HA‐tagged candidate constructs. Co‐immunoprecipitation (Co‐IP) assays showed that β‐adducin interacted with PLA2G4A and IMPACT, but not with Dpp3. Subsequent reciprocal Co‐IP further confirmed that β‐adducin interacts with both PLA2G4A and IMPACT (Figure 4C,D). Given the reported role of PLA2G4A in lysosomal membrane damage, we focused on PLA2G4A in subsequent experiments. The interaction between endogenous β‐adducin and PLA2G4A was further confirmed in primary cortical neurons via Co‐IP and immunofluorescence staining (Figure 4E; Figure S7A). Importantly, endogenous Co‐IP also demonstrated their association in ischemic cortical tissue collected at 3 days after MCAO (Figure S7B).

**FIGURE 4:**
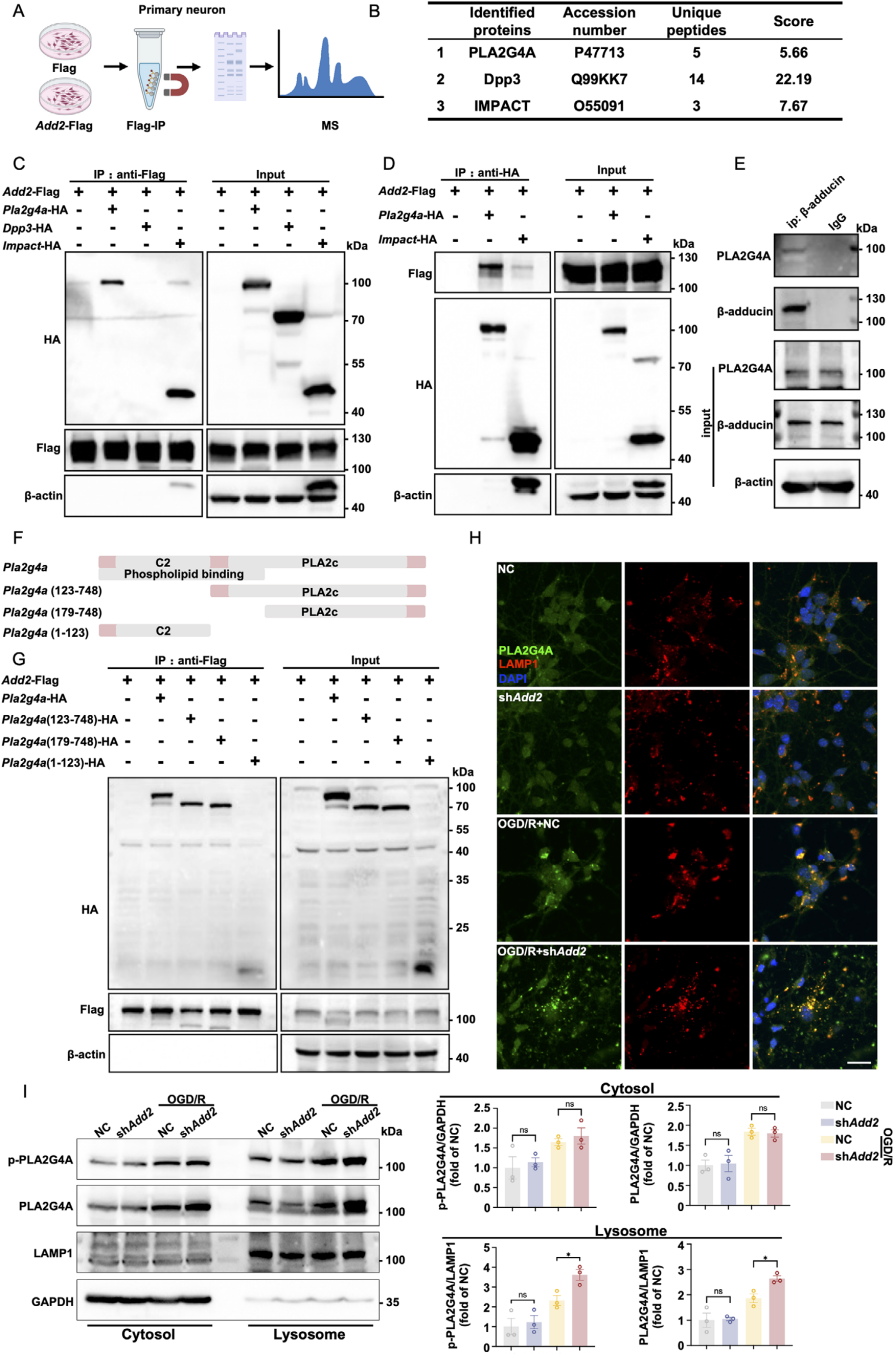
β‐adducin interacts with PLA2G4A and limits its lysosomal accumulation during OGD/R. (A) Schematic representation of the immunoprecipitation‐mass spectrometry (IP‐MS) screening strategy. (B) List of candidate β‐adducin‐interacting proteins identified in primary neurons. (C,D) Co‐IP assays validating the interactions between β‐adducin and the candidate proteins in HEK293T cells. Cells were co‐transfected with *Add2*‐Flag and HA‐tagged candidate plasmids. The protein interactions were confirmed by immunoprecipitation with an anti‐Flag antibody (C) or an anti‐HA antibody (D). (E) Co‐IP assay demonstrating the endogenous association between β‐adducin and PLA2G4A in primary neurons. IgG served as the negative control. (F) Schematic diagram of full‐length PLA2G4A and its specific truncation mutants. (G) Co‐IP assay revealing that β‐adducin associates with multiple PLA2G4A regions. (H) Immunostaining for PLA2G4A (green) and the lysosomal marker LAMP1 (red) in primary neurons after *Add2* silencing. Scale bar, 20 µm. (I) Subcellular fractionation and Western blot analysis showing the distribution of p‐PLA2G4A and PLA2G4A within the cytosolic and lysosomal fractions (*n* = 3, one‐way ANOVA, *p < 0.05, ns, not significant). GAPDH and LAMP1 were used as specific markers for the cytosolic and lysosomal fractions, respectively. Data are expressed as the mean ± SEM. The original blot can be found in Figure S16.

To further characterize the interaction between β‐adducin and PLA2G4A, we generated a series of truncated PLA2G4A constructs (Figure 4F). PLA2G4A contains an N‐terminal C2 domain involved in calcium‐dependent membrane binding and a C‐terminal catalytic domain responsible for phospholipid hydrolysis. Co‐IP analysis showed that β‐adducin interacted with both the N‐terminal and C‐terminal regions of PLA2G4A, suggesting that the association was not restricted to a single region (Figure 4G). Additional Co‐IP analysis showed that phosphorylated PLA2G4A was also associated with β‐adducin (Figure S7C). Notably, the N‐terminal PLA2G4A (1–123) fragment, which lacks the canonical Ser505 phosphorylation site, retained its association with β‐adducin, indicating that the association can occur in the absence of the Ser505‐containing region. Moreover, β‐adducin remained associated with PLA2G4A under OGD conditions both with and without BAPTA‐AM, a cell‐permeable Ca^2^^+^ chelator (Figure S7D), suggesting that intracellular Ca^2^^+^ elevation did not abolish the association.

We next examined whether β‐adducin affects the subcellular redistribution of PLA2G4A during ischemic stress. Immunofluorescence staining showed that OGD/R increased the colocalization of PLA2G4A with the lysosomal marker LAMP1 in primary neurons, and this effect was further enhanced by β‐adducin knockdown (Figure 4H). Consistently, western blot analysis of isolated lysosomal fractions showed that both total and phosphorylated PLA2G4A were enriched in lysosomes after OGD/R, and this enrichment was further increased in sh*Add2*‐treated neurons (Figure 4I). These results indicate that β‐adducin interacts with PLA2G4A and limits its lysosomal accumulation during ischemic injury.

To further determine whether β‐adducin‐mediated regulation of PLA2G4A involves changes in its intrinsic activation state, we examined total PLA2G4A expression, phosphorylation, and enzymatic activity following β‐adducin manipulation. Neither β‐adducin knockdown nor overexpression altered total PLA2G4A abundance or the p‐PLA2G4A/PLA2G4A ratio under basal or OGD/R conditions (Figure S8A,B). Consistently, β‐adducin manipulation did not significantly affect PLA2G4A enzymatic activity in the cytosolic fraction. However, under OGD/R conditions, *Add2* knockdown increased PLA2G4A activity in the lysosomal fraction, whereas *Add2* overexpression reduced lysosome‐associated PLA2G4A activity (Figure S8C,D). These findings suggest that β‐adducin primarily regulates the pathological redistribution of PLA2G4A to lysosomal membranes rather than directly modifying its intrinsic catalytic activation.

### 2.5 β‐Adducin Limits PLA2G4A‐Associated Lysosomal Injury and Ischemic Brain Damage

Given the increased lysosomal accumulation of PLA2G4A after β‐adducin loss, we next investigated whether this redistribution contributes to LMP during ischemic stress. EGFP‐LGALS3 puncta, a marker of damaged lysosomal membranes, were rarely detected under basal conditions but increased markedly following OGD/R. β‐adducin knockdown further increased LGALS3 puncta formation, whereas simultaneous *Pla2g4a* knockdown significantly attenuated this effect (Figure 5A,B). Efficient double knockdown of *Add2* and *Pla2g4a* was confirmed by qPCR and western blotting (Figure S9A,B), supporting the involvement of PLA2G4A in the enhanced lysosomal membrane damage caused by β‐adducin deficiency.

**FIGURE 5:**
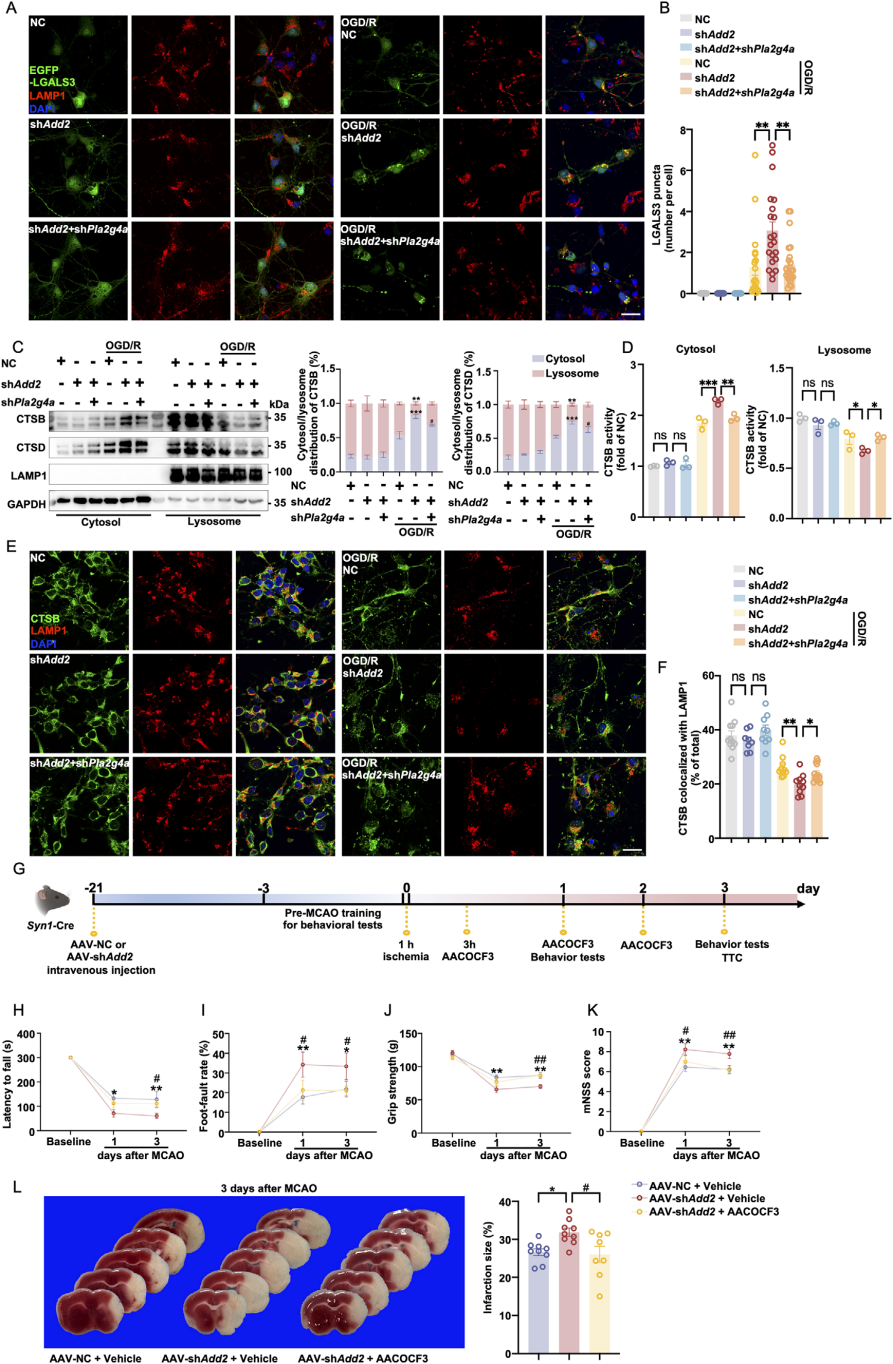
β‐adducin limits PLA2G4A‐associated lysosomal injury and ischemic brain damage. (A,B) Representative immunofluorescence images (A) and quantification (B) of LAMP1‐positive EGFP‐LGALS3 puncta in primary neurons with the indicated treatments (*n* = 3, one‐way ANOVA, **p < 0.01). EGFP‐LGALS3 is shown in green, LAMP1 in red, and nuclei in blue. Scale bar, 20 µm. (C) Subcellular fractionation analysis confirming that PLA2G4A silencing attenuates the OGD/R‐induced cytosolic redistribution of CTSB and CTSD to the cytosolic fraction exacerbated by *Add2* knockdown (*n* = 3, one‐way ANOVA, **p < 0.01, ***p < 0.001 vs. OGD/R+NC, ^#^ *p* < 0.05 vs. OGD/R+sh*Add2*). (D) Measurement of CTSB activity in the cytosolic and lysosomal fractions under the indicated condition (*n* = 3, one‐way ANOVA, *p < 0.05, **p < 0.01, ***p < 0.001, ns, not significant). (E,F) Immunofluorescence images (E) and quantification (F) of the colocalization of CTSB (green) with LAMP1 (red) in primary neurons (*n* = 3, one‐way ANOVA, *p < 0.05, **p < 0.01, ns, not significant). Scale bar, 20 µm. (G) Schematic illustration of the experimental design and AACOCF3 treatment schedule. (H–K) Neurological function assessed by the rotarod test (H), foot‐fault rate (I), grip strength (J), and mNSS score (K) at baseline and at 1 and 3 days after MCAO (*n* = 9 for AAV‐NC + vehicle and AAV‐sh*Add2* + vehicle, *n* = 8 for AAV‐sh*Add2 *+ AACOCF3, two‐way ANOVA, *p < 0.05, **p < 0.01 vs. AAV‐NC + vehicle; ^#^ *p *< 0.05, ^##^ *p* < 0.01 vs. AAV‐sh*Add2 *+ vehicle). (L) Representative TTC‐stained brain sections and quantification of cerebral infarct volume at 3 days after MCAO (*n* = 9 for AAV‐NC + vehicle and AAV‐sh*Add2* + vehicle, *n* = 8 for AAV‐sh*Add2* + AACOCF3, one‐way ANOVA, *p < 0.05 vs. AAV‐NC + vehicle; ^#^ *p* < 0.05 vs. AAV‐sh*Add2* + vehicle). Data are expressed as the mean ± SEM. The original blot can be found in Figure S17.

We therefore examined the subcellular distribution of Cathepsin B (CTSB) and Cathepsin D (CTSD), two lysosomal proteases whose cytosolic redistribution reflects lysosomal membrane damage [33]. Subcellular fractionation showed that OGD/R increased the redistribution of CTSB and CTSD to the cytosolic fraction, accompanied by reduced retention in the lysosomal fraction. These changes were further enhanced by *Add2* knockdown and partially reversed by simultaneous *Pla2g4a* knockdown (Figure 5C). In parallel, CTSB enzymatic activity was increased in the cytosol and reduced in the lysosomal fraction following OGD/R. β‐adducin knockdown further increased cytosolic CTSB activity, whereas *Pla2g4a* knockdown attenuated these changes (Figure 5D). Immunofluorescence analysis similarly showed reduced CTSB/LAMP1 colocalization after OGD/R, which was further decreased by *Add2* knockdown and partially restored by *Pla2g4a* knockdown (Figure 5E,F). Comparable changes were observed for CTSD/LAMP1 colocalization (Figure S9C,D). LysoSensor and LysoTracker analyses further showed impaired lysosomal acidification and homeostasis after OGD/R, with greater alterations following *Add2* knockdown and partial recovery after *Pla2g4a* knockdown (Figure S9E,F).

To exclude potential sequence‐specific effects of *Add2* knockdown, we repeated the key experiments using a second, non‐overlapping *Add2* shRNA. This independent shRNA produced a similar pattern of increased PLA2G4A/LAMP1 colocalization and also enhanced LGALS3 puncta formation, cytosolic CTSB activity, and neuronal death following OGD/R (Figure S10), further supporting the specificity of the β‐adducin loss‐of‐function phenotype.

To determine whether β‐adducin deficiency also aggravates lysosomal injury in vivo, cytosolic and lysosomal fractions were isolated from the ischemic penumbra 3 days after MCAO. MCAO increased the redistribution of CTSD from the lysosomal to the cytosolic fraction, and neuronal *Add2* knockdown further enhanced this shift compared with AAV‐NC mice (Figure S11A,B). These findings extend our in vitro observations to the ischemic brain and are consistent with enhanced lysosomal protease leakage following neuronal β‐adducin deficiency in vivo.

We next examined whether pharmacological inhibition of PLA2G4A could attenuate the aggravated ischemic injury associated with β‐adducin deficiency in vivo. Neuron‐specific *Add2*‐knockdown mice were subjected to MCAO, and AACOCF3 treatment was initiated 3 h after MCAO, followed by additional administration on days 1 and 2 (Figure 5G). AACOCF3 treatment reduced PLA2G4A enzymatic activity in AAV‐sh*Add2* mice compared with vehicle‐treated controls (Figure S11C). Importantly, AACOCF3 attenuated the neurological deficits associated with β‐adducin knockdown, as assessed by the rotarod performance, foot‐fault rate, grip strength, and mNSS score (Figure 5H–K). Consistent with these functional improvements, AACOCF3 treatment also reduced cerebral infarct volume at 3 days after MCAO (Figure 5L).

Collectively, these findings indicate that β‐adducin deficiency enhances PLA2G4A‐associated lysosomal membrane damage and protease leakage during ischemic stress. Genetic suppression of PLA2G4A attenuates these lysosomal abnormalities in vitro, whereas pharmacological inhibition of PLA2G4A mitigates the aggravated ischemic brain injury associated with β‐adducin deficiency in vivo.

### 2.6 FTO Restores β‐Adducin Expression and Protects Against Ischemic Brain Injury

Having established the neuroprotective role of β‐adducin, we next investigated the upstream mechanisms responsible for its reduction after ischemic injury. Because the brain‐specific *Add2* isoform contains an extended 3' UTR that may be susceptible to post‐transcriptional regulation, we examined whether m^6^A modification is involved [25]. Dot blot analysis showed that global m^6^A levels were increased in the ischemic penumbra at 1, 3, and 7 days after MCAO (Figure 6A). MeRIP‐qPCR further showed increased m^6^A enrichment on *Add2* mRNA at 1 and 3 days post‐MCAO (Figure 6B).

**FIGURE 6:**
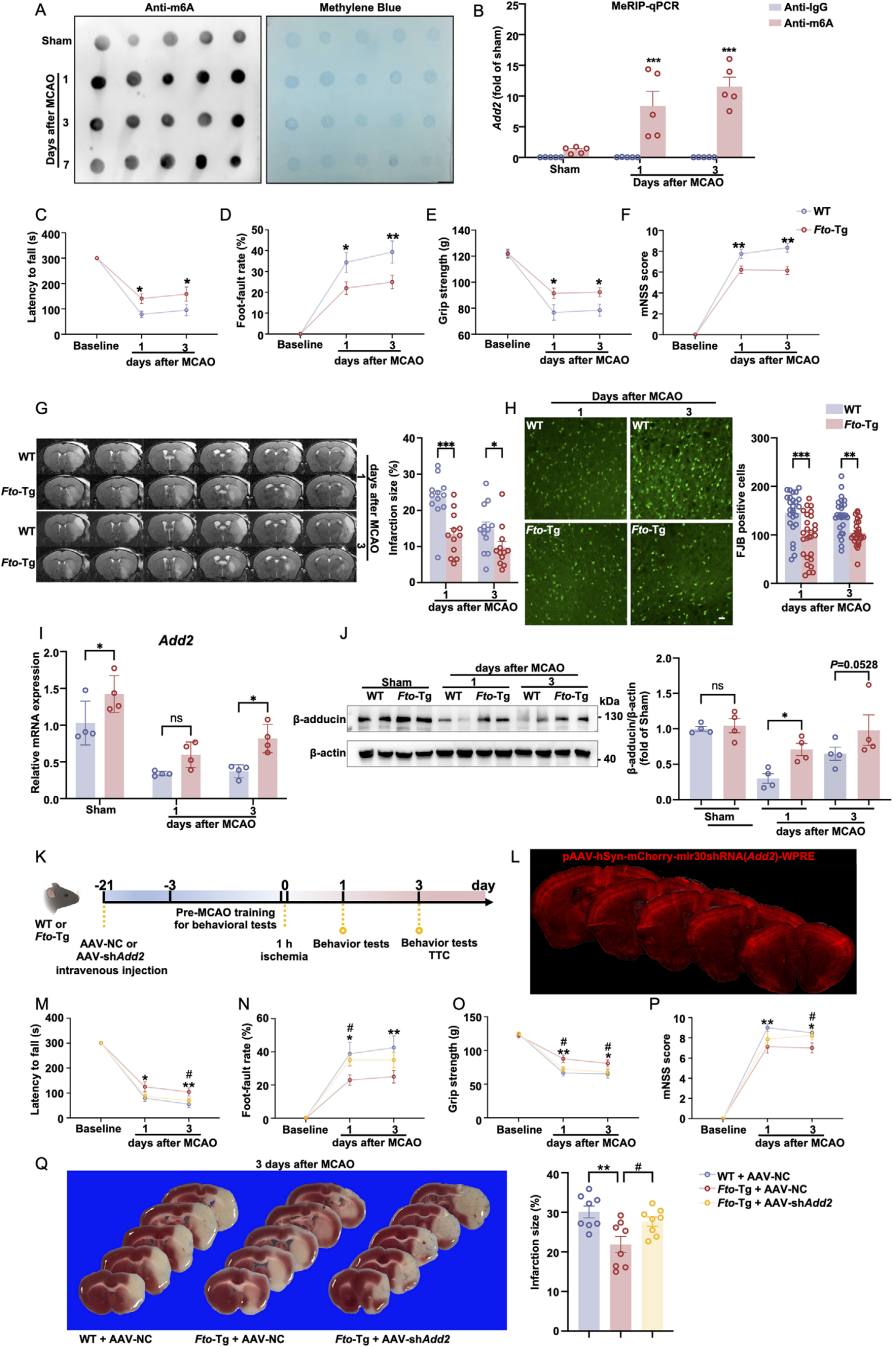
FTO restores β‐adducin expression and protects against ischemic brain injury. (A) Dot blot analysis revealing the global m^6^A RNA methylation levels in the ischemic penumbra at 1, 3, and 7 days following MCAO compared to the sham group. (B) MeRIP‐qPCR assay evaluating the specific m^6^A enrichment on the *Add2* transcript at 1 and 3 days after MCAO (*n* = 5, two‐way ANOVA, ***p < 0.001). (C–F) Neurological function was assessed in wild‐type (WT) and *Fto* transgenic (*Fto*‐Tg) mice according to the latency to fall in the rotarod test (C), foot‐fault rate (D), grip strength (E), and mNSS score (F) at 1 and 3 days after MCAO (*n* = 12 for WT, *n* = 13 for *Fto*‐Tg, two‐way ANOVA, *p < 0.05, **p < 0.01). (G) Representative *T*_2_‐weighted MRI images and quantitative analysis of cerebral infarct volume in WT and *Fto*‐Tg mice (*n* = 12, two‐way ANOVA, *p < 0.05, ***p < 0.001). (H) FJB staining and the corresponding quantitative analysis of FJB‐positive degenerating neurons within the ischemic penumbra (*n* = 4, two‐way ANOVA, **p < 0.01, ***p < 0.001). Scale bar, 20 µm. (I) Relative *Add2* mRNA levels in WT and *Fto*‐Tg mice (*n* = 4, two‐way ANOVA, *p < 0.05, ns, not significant). (J) Protein levels of β‐adducin relative to β‐actin in WT and *Fto‐*Tg mice (*n* = 4, two‐way ANOVA, *p < 0.05, ns, not significant). (K) Schematic illustration of the experimental design. (L) Representative whole‐brain mCherry fluorescence images showing the cerebral distribution of the AAV‐derived mCherry signal following AAV‐sh*Add2* administration. (M–P) Neurological function assessed by the rotarod test (M), foot‐fault rate (N), grip strength (O), and mNSS score (P) at baseline and at 1 and 3 days after MCAO (*n* = 8, two‐way ANOVA, *p < 0.05, **p < 0.01 vs. WT + AAV‐NC; ^#^ *p* < 0.05, ^##^ *p* < 0.01 vs. *Fto‐*Tg + AAV‐NC). (Q) Representative TTC‐stained brain sections and quantification of cerebral infarct volume at 3 days after MCAO (*n* = 8, one‐way ANOVA, **p < 0.01 vs. WT + AAV‐NC; ^#^ *p* < 0.05 vs. *Fto‐*Tg + AAV‐NC). Data are expressed as the mean ± SEM. The original blot can be found in Figure S17.

To identify candidate regulators associated with this hypermethylation, we analyzed the expression of major m^6^A writers (*Mettl3*, *Mettl14*, *Wtap*) and erasers (*Fto*, *Alkbh5*). Although *Mettl3* and *Mettl14* were reduced, FTO expression was also markedly decreased, suggesting that impaired m^6^A demethylation may contribute to the increased m^6^A modification of *Add2* mRNA (Figure S12A).

To establish the role of FTO‐mediated β‐adducin regulation in vivo, we subjected *Fto* transgenic (*Fto*‐Tg) mice to the MCAO model (Figure S12B–D). Neurological outcomes were evaluated at 1 and 3 days after MCAO. Compared with wild‐type (WT) controls, FTO overexpression ameliorated ischemia‐induced neurobehavioral deficits, as evidenced by significant improvements in the rotarod test, foot‐fault test, grip strength, and mNSS scores (Figure 6C–F). Consistent with these functional improvements, T2‐weighted MRI revealed a reduction in cerebral infarct volume in *Fto‐*Tg mice (Figure 6G). Furthermore, FJB staining revealed fewer degenerating neurons in the ischemic penumbra (Figure 6H). Importantly, qPCR and western blot analyses showed that FTO overexpression restored β‐adducin expression in the ischemic penumbra at 1 and 3 days after MCAO (Figure 6I,J). These findings indicate that FTO overexpression alleviates acute ischemic brain injury and is accompanied by restoration of β‐adducin expression.

To determine whether β‐adducin contributes to the neuroprotective effects of FTO in vivo, we knocked down neuronal *Add2* in *Fto*‐Tg mice using an hSyn‐driven AAV‐sh*Add2* administered three weeks before MCAO (Figure 6K). Whole‐brain mCherry fluorescence confirmed widespread cerebral distribution of the AAV vector (Figure 6L), while neuronal targeting and efficient *Add2* knockdown were further validated by cell‐type‐specific immunostaining, qPCR, and western blotting (Figure S12E–G). Importantly, *Add2* knockdown did not alter the elevated FTO expression in *Fto*‐Tg mice. Functionally, neuronal *Add2* knockdown significantly weakened the neurological benefits conferred by FTO overexpression, as demonstrated by poorer performance in the rotarod, foot‐fault, grip strength, and mNSS tests after MCAO (Figure 6M–P). Consistently, TTC staining showed that the reduction in cerebral infarct volume observed in *Fto*‐Tg mice was partially reversed by neuronal *Add2* knockdown (Figure 6Q). Together, these findings indicate that β‐adducin contributes substantially to FTO‐mediated neuroprotection in vivo, supporting a functional FTO‐β‐adducin axis in ischemic stroke.

### 2.7 FTO Stabilizes *Add2* mRNA Through m^6^A Demethylation and Protects Neurons Against OGD/R‐Induced Injury

To clarify how FTO regulates β‐adducin under ischemic conditions, we next used an in vitro OGD/R model. Consistent with the in vivo findings, dot blot analysis revealed increased global m^6^A levels at 3, 6, 12, and 24 h following OGD/R. qPCR analysis further showed that *Fto* expression was markedly reduced, whereas *Alkbh5* expression was increased and the methyltransferases *Mettl3*, *Mettl14*, and *Wtap* remained largely unchanged (Figure S13A,B). Furthermore, MeRIP‐qPCR confirmed increased m^6^A enrichment on *Add2* mRNA after OGD/R treatment (Figure 7A).

**FIGURE 7:**
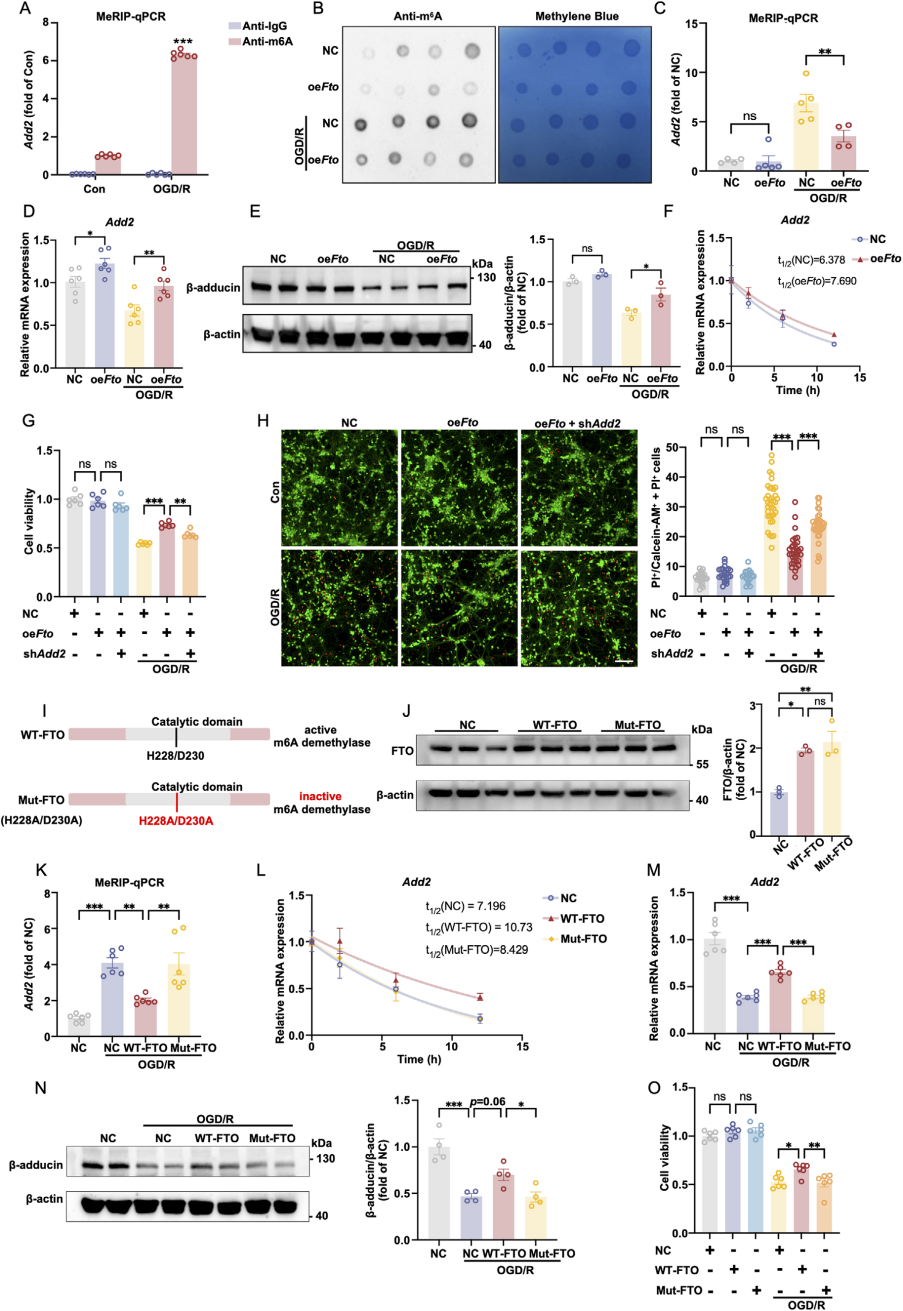
FTO stabilizes *Add2* mRNA through m^6^A demethylation and protects neurons against OGD/R‐induced injury. (A) MeRIP‐qPCR assay assessing the m^6^A enrichment on the *Add2* transcript in primary neurons subjected to OGD/R (*n* = 6, two‐way ANOVA, ***p < 0.001). (B) Dot blot assay evaluating global m^6^A RNA methylation levels following *Fto* overexpression (oe*Fto*). (C) MeRIP‐qPCR analysis of m^6^A modification on *Add2* mRNA following FTO overexpression under OGD/R conditions (*n* = 4 to 5, one‐way ANOVA, **p < 0.01, ns, not significant). (D) Relative *Add2* mRNA level in primary neurons after *Fto* overexpression (*n* = 6, one‐way ANOVA, *p < 0.05, **p < 0.01). (E) Western blot analysis of β‐adducin expression in primary neurons after *Fto* overexpression (*n* = 3, one‐way ANOVA, *p < 0.05). (F) mRNA stability assay evaluating the decay and half‐life of the *Add2* transcript in primary neurons expressing NC or oe*Fto* following transcriptional inhibition (*n* = 6, non‐linear regression curve fitting). (G) Assessment of cell viability in primary neurons across the indicated experimental groups following OGD/R exposure (*n* = 6, one‐way ANOVA, **p < 0.01, ***p < 0.001). (H) Calcein‐AM/PI staining images and the quantification of PI‐positive dead cells in the indicated experimental groups (*n* = 3, one‐way ANOVA, ***p < 0.001, ns, not significant). Scale bar, 100 µm. (I) Schematic illustration of wild‐type FTO (WT‐FTO) and the catalytically inactive FTO mutant carrying the H228A/D230A substitutions (Mut‐FTO). (J) Representative Western blot images and quantitative analysis of FTO protein expression in primary cortical neurons transduced with NC, WT‐FTO, or Mut‐FTO (*n* = 3, one‐way ANOVA, *p < 0.05, **p < 0.01). (K) MeRIP‐qPCR analysis of m^6^A enrichment on *Add2* mRNA in the indicated groups under basal and OGD/R conditions (*n* = 6, one‐way ANOVA, **p < 0.01, ***p < 0.001). (L) mRNA stability assay showing *Add2* transcript decay following transcriptional inhibition in neurons expressing NC, WT‐FTO, or Mut‐FTO (*n* = 6, non‐linear regression curve fitting). (M) qPCR analysis of *Add2* mRNA expression in neurons expressing NC, WT‐FTO, or Mut‐FTO under the indicated conditions (*n* = 6, one‐way ANOVA, ***p < 0.001). (N) Representative western blots and quantitative analysis of β‐adducin protein expression in neurons expressing NC, WT‐FTO, or Mut‐FTO following OGD/R (*n* = 4, one‐way ANOVA, *p < 0.05, ***p < 0.001). (O) Cell viability measured by CCK‐8 assay in neurons expressing NC, WT‐FTO, or Mut‐FTO under basal and OGD/R conditions (*n* = 6, one‐way ANOVA, *p < 0.05, **p < 0.01). Data are expressed as the mean ± SEM. The original blot can be found in Figure S17.

To determine whether FTO regulates these m^6^A changes, we overexpressed FTO (oe*Fto*) in primary neurons (Figure S13C,D). Remarkably, oe*Fto* not only attenuated the global m^6^A elevation under both normoxic and OGD/R conditions but also reduced m^6^A modifications on *Add2* mRNA (Figure 7B,C). FTO overexpression also increased β‐adducin expression at both the mRNA and protein levels (Figure 7D,E). Given that *Add2* mRNA abundance was altered, we next assessed transcript stability. RNA stability assays showed that oe*Fto* prolonged the half‐life of *Add2* mRNA, supporting a role for FTO in stabilizing the *Add2* transcript (Figure 7F).

To determine whether β‐adducin contributes to FTO‐mediated neuroprotection, we co‐transfected primary neurons with oe*Fto* and sh*Add2*. The efficacy of this dual‐modulation strategy was confirmed at both the mRNA and protein levels (Figure s13 E–G). CCK‐8 and Calcein‐AM/PI staining showed that FTO overexpression markedly protected primary neurons from OGD/R‐induced death; however, this neuroprotective effect was partially weakened by the concurrent knockdown of β‐adducin (Figure 7G,H). These data support a role for FTO in promoting *Add2* mRNA stability and indicate that β‐adducin contributes to FTO‐mediated protection against ischemic neuronal injury.

To further determine whether FTO‐mediated regulation of *Add2* requires its m^6^A demethylase activity, a catalytically inactive FTO mutant carrying the H228A/D230A substitutions (Mut‐FTO) was generated and compared with wild‐type FTO (WT‐FTO) (Figure 7I). WT‐FTO and Mut‐FTO were expressed at comparable protein levels in primary neurons (Figure 7J). Following OGD/R, WT‐FTO reduced both global m^6^A levels and m^6^A enrichment on *Add2* mRNA, whereas Mut‐FTO failed to produce comparable reductions (Figure 7K; Figure s13 H). WT‐FTO also prolonged the half‐life of *Add2* mRNA, whereas Mut‐FTO did not reproduce this stabilizing effect (Figure 7L). Consistently, WT‐FTO restored *Add2* mRNA and β‐adducin protein expression following OGD/R, whereas Mut‐FTO failed to reproduce these effects (Figure 7M,N). WT‐FTO also improved neuronal viability after OGD/R, while Mut‐FTO failed to confer comparable protection (Figure 7O).

We further examined whether FTO overexpression affects PLA2G4A expression, subcellular localization, and lysosomal integrity following OGD/R. Although OGD/R increased *Pla2g4a* mRNA and total PLA2G4A protein levels, FTO overexpression did not significantly alter either measure (Figure S14A,B). In contrast, FTO overexpression reduced PLA2G4A accumulation in the lysosomal fraction without significantly affecting its cytosolic abundance, accompanied by an apparent reduction in PLA2G4A/LAMP1 colocalization under OGD/R conditions (Figure S14C,D). FTO overexpression also reduced LGALS3 puncta formation and attenuated the redistribution of lysosomal cathepsins from the lysosomal to the cytosolic fraction after OGD/R (Figure S14E–G). These findings indicate that FTO overexpression is associated with reduced lysosomal redistribution of PLA2G4A and preservation of lysosomal integrity, rather than changes in overall PLA2G4A expression.

Together, these findings suggest that FTO‐mediated stabilization of *Add2* mRNA and restoration of β‐adducin expression require the m^6^A demethylase activity of FTO, and further support β‐adducin as a downstream contributor to FTO‐mediated neuroprotection during ischemic stress.

## 3 Discussion

In the present study, we identify β‐adducin as an endogenous protector of lysosomal integrity in ischemic neurons. Our data support a model in which ischemic stress reduces FTO expression, increases m^6^A modification on *Add2* mRNA, and accelerates *Add2* transcript decay, thereby lowering β‐adducin levels. The resulting loss of β‐adducin is associated with increased lysosomal accumulation of PLA2G4A, enhanced LMP, cathepsin leakage, and neuronal death. Conversely, restoring β‐adducin expression or interrupting the PLA2G4A‐associated injury pathway confers neuroprotection in vitro and in vivo (Figure 8). Thus, our study suggests that β‐adducin is an important component of an intrinsic lysosomal defense mechanism that helps preserve neuronal viability under ischemic stress.

**FIGURE 8:**
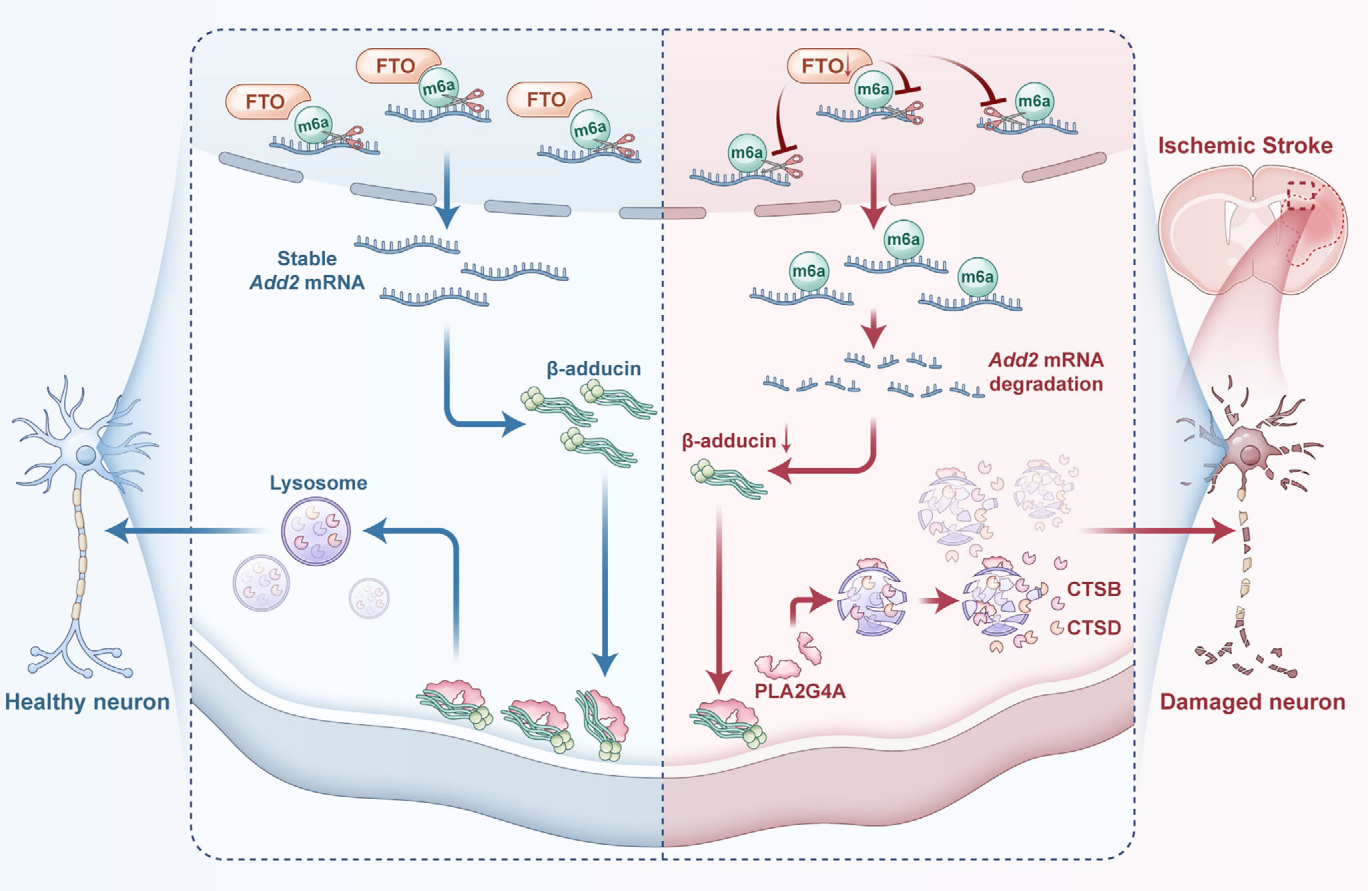
Reduced FTO‐mediated demethylation promotes β‐adducin loss, thereby promoting PLA2G4A‐associated lysosomal injury and neuronal death following ischemic stroke. Under physiological conditions, FTO‐mediated m^6^A demethylation helps maintain *Add2* mRNA stability, thereby sustaining β‐adducin expression in neurons and limiting lysosomal accumulation of PLA2G4A to maintain lysosomal integrity. Under ischemic conditions, reduced FTO expression is associated with increased m^6^A modification of *Add2* mRNA, accelerated *Add2* transcript degradation, and decreased β‐adducin expression. Loss of β‐adducin promotes lysosomal accumulation of PLA2G4A, leading to lysosomal membrane permeabilization, cytosolic leakage of cathepsins, including CTSB and CTSD, and ultimately neuronal injury.

Adducins are membrane‐associated actin‐binding proteins that cap actin filaments and promote the assembly of the spectrin‐actin network [34, 35]. Unlike the ubiquitously expressed α‐ and γ‐isoforms, β‐adducin exhibits profound tissue specificity, being restricted to erythrocytes and the nervous system. In the periphery, genetic ablation or mutation of β‐adducin disrupts the membrane‐skeleton network and perturbs Na/K‐ATPase activity, thereby precipitating the pathogenesis of primary hypertension and spherocytic hereditary elliptocytosis [20, 36, 37]. In the central nervous system, β‐adducin regulates synapse formation and stability, serving as a physical substrate for hippocampal LTP and long‐term memory consolidation [22, 23, 38]. Against this background, our findings extend the functional profile of β‐adducin beyond its established structural role. Rather than acting solely as a structural membrane‐associated protein, β‐adducin appears to participate in the preservation of subcellular membrane stability during pathological stress. In the setting of ischemic injury, where lysosomal disruption is increasingly recognized as a determinant of irreversible neuronal damage, this previously unappreciated function of β‐adducin may be particularly important [9, 39, 40]. β‐adducin is predominantly enriched in neurons and contributes to neuronal survival following ischemic stroke. Mechanistically, the interaction between β‐adducin and PLA2G4A appears to contribute substantially to this endogenous protection. We postulate that β‐adducin functions as a cytosolic constraint for PLA2G4A.

PLA2G4A is a phospholipid hydrolase whose cleavage products—free fatty acids and phospholipids—can promote oxidative stress and inflammatory signaling [41]. Mounting evidence indicates that PLA2G4A‐mediated phospholipid cleavage acts as a critical step in triggering the physical collapse of organelles. In traumatic brain injury and spinal cord injury, hyperactive PLA2G4A drives the occurrence of LMP by directly depriving neuronal lysosomes of their membrane fluidity and permeability homeostasis, ultimately resulting in the total paralysis of membrane integrity and the lethal release of internal enzymes into the cytoplasm [17, 42]. In line with these established mechanisms, our subcellular fractionation analysis reveals that ischemic injury triggers a marked accumulation of PLA2G4A in the lysosomal fraction, a phenomenon that is significantly aggravated following the loss of β‐adducin. These findings place PLA2G4A at an important junction between ischemic stress and lysosomal damage. Importantly, our additional analyses indicate that β‐adducin does not substantially alter total PLA2G4A expression or overall PLA2G4A phosphorylation, but primarily regulates its subcellular distribution. Loss of β‐adducin increases lysosome‐associated PLA2G4A accumulation and activity without altering cytosolic PLA2G4A activity, supporting a model in which β‐adducin preserves lysosomal integrity by limiting PLA2G4A accumulation at lysosomes rather than by directly inhibiting its intrinsic catalytic function.

The function of PLA2G4A depends on its C2 domain and the Ser505‐phosphorylation‐regulated enzymatic region [43, 44, 45]. Our truncation analysis further indicates that the interaction between β‐adducin and PLA2G4A is not restricted to a single minimal binding region. Although we initially considered the possibility that β‐adducin might primarily interfere with the C2 domain, PLA2G4A mutants lacking either the C2 domain or the phospholipid‐binding region retained the capacity to interact with β‐adducin, and the isolated C2 domain was also sufficient for binding. Notably, the 1–123 fragment, which lacks the canonical Ser505 phosphorylation site, retained β‐adducin binding, whereas phosphorylated PLA2G4A was also detected in β‐adducin immunoprecipitates. These findings suggest that Ser505 phosphorylation is not strictly required for the association. Moreover, the β‐adducin‐PLA2G4A association remained detectable under both OGD conditions and intracellular Ca^2^^+^ chelation with BAPTA‐AM, indicating that their association can be maintained across different intracellular Ca^2^^+^ conditions. Taken together, these observations suggest that β‐adducin engages PLA2G4A through multiple interfaces, a configuration that may help limit its pathological redistribution to lysosomes during ischemic stress. At present, however, our data support an interaction‐based restriction model rather than a definitive steric hindrance mechanism, and the precise structural basis of this restraint will require further investigation.

As the central catabolic hub of the cell, the lysosome is enriched with active acidic hydrolases and iron. While mild, early‐stage LMP can initiate compensatory repair programs, lethal LMP triggered by severe ischemic stress irreversibly compromises this physical barrier. Upon LMP, lysosomal cathepsins and redox‐active iron can gain access to the cytosol, promoting proteolytic and oxidative injury and contributing to multiple forms of cell death [33, 46, 47, 48]. In this context, our data indicate that β‐adducin‐dependent regulation of PLA2G4A has important consequences for lysosomal integrity and downstream neuronal death signaling. Our data demonstrate that ischemic injury provokes cytosolic redistribution of the lysosomal proteases CTSB and CTSD, accompanied by increased cleavage of Caspase‐9 and Caspase‐3 [49, 50]. β‐Adducin knockdown significantly amplifies cathepsin redistribution and apoptotic signaling, whereas its overexpression attenuates these changes. More importantly, simultaneous knockdown of β‐adducin and PLA2G4A attenuates the exacerbation induced by *Add2* knockdown alone, supporting an important role for PLA2G4A in the lysosomal injury associated with β‐adducin deficiency. Notably, many lysosomal hydrolases exhibit reduced activity after release into the near‐neutral cytosolic environment [51]. Liu et al. found that cytosolic CTSB can nevertheless retain substantial catalytic activity at neutral pH [52]. Our enzymatic assays determined that CTSB activity is increased in the cytosol following OGD/R. Moreover, β‐adducin knockdown further enhances this aberrant cytosolic CTSB activity, which is attenuated by simultaneous PLA2G4A knockdown. Together, these findings indicate that β‐adducin limits PLA2G4A‐associated lysosomal injury, cathepsin leakage, and downstream apoptotic signaling in ischemic neurons.

Having established the critical role of β‐adducin in preventing LMP, we next sought to elucidate the upstream mechanisms driving its rapid decrease following ischemic injury. Notably, we also observed a decline in α‐adducin. Previous studies have demonstrated that α‐adducin requires heterologous binding partners for its stability [53]. Accordingly, we hypothesize that the observed reduction in α‐adducin may be secondary to the β‐adducin loss, although this possibility requires further investigation. Brain‐specific β‐adducin mRNA transcripts are unusually long due to a tissue‐specific alternative polyadenylation mechanism that generates a massive 3' UTR [25]. This extensive 3' UTR renders the *Add2* mRNA highly susceptible to post‐transcriptional modifications, and m^6^A represents the most abundant internal modification of mammalian mRNA [25, 54]. Extensive evidence from clinical cohorts and diverse preclinical stroke models has established that ischemic injury triggers increased global RNA m^6^A modification [26, 28, 55]. In line with these observations, our dot blot analysis verified a significant accumulation of global m^6^A levels following ischemic challenge in our experimental models. While the literature presents conflicting views on whether this hypermethylation is driven by the upregulation of methyltransferases (e.g., METTL3, METTL14) or the downregulation of demethylases [28, 56, 57, 58], our data revealed a concurrent decrease in the expression of the m^6^A writers METTL3/METTL14 and the demethylase FTO. These findings suggest that reduced FTO‐mediated demethylation contributes to the increased m^6^A modification of *Add2* in our models, although additional regulation by m^6^A writers or readers cannot be excluded. Functionally, m^6^A modifications dictate RNA fate depending on the recruitment of specific reader proteins [59, 60, 61]. Because FTO overexpression increased *Add2* mRNA levels, we examined transcript stability and found that FTO overexpression prolonged the half‐life of *Add2* mRNA. Importantly, the catalytically inactive H228A/D230A FTO mutant failed to reproduce the effects of WT‐FTO on *Add2* m^6^A modification, mRNA stability, and β‐adducin expression, indicating that these effects require FTO demethylase activity. Consistent with this model, FTO overexpression did not alter total PLA2G4A expression but reduced its lysosomal accumulation, LGALS3 puncta formation, and lysosomal cathepsin redistribution following OGD/R, further linking FTO activity to preservation of lysosomal integrity. The functional relevance of this pathway was further supported in vivo, where neuronal *Add2* knockdown substantially weakened the neuroprotective effects of FTO upregulation in *Fto*‐Tg mice after MCAO. Although FTO undoubtedly regulates additional m^6^A‐modified transcripts, these findings identify β‐adducin as an important downstream mediator of FTO‐mediated neuroprotection in ischemic stroke. Thus, our data support a model in which ischemia‐associated FTO loss contributes to increased m^6^A modification of *Add2* mRNA, reduces its stability, and consequently decreases β‐adducin expression. Although the exact m^6^A reader remains uncharacterized, the present findings place FTO loss‐associated *Add2* destabilization upstream of the β‐adducin/PLA2G4A/LMP axis.

While a mechanistic framework for β‐adducin‐mediated lysosomal protection has been established, several limitations warrant further investigation. First, although our data indicate that reduced FTO‐mediated demethylation contributes to increased m^6^A modification and destabilization of *Add2* mRNA, the specific m^6^A reader protein responsible for the transcript decay remains unidentified. Accordingly, although our data support a role for m^6^A‐dependent *Add2* destabilization, the complete post‐transcriptional regulatory machinery remains to be defined. Second, our experiments concentrated on the neuron‐enriched β‐adducin. Although we postulate that the observed decline in α‐adducin may be a secondary consequence of β‐adducin loss, its distinct functional contributions, as well as the roles of adducin complexes in other neural cell types, require further elucidation. Third, although reduced β‐adducin expression was observed in human postmortem ischemic stroke tissues, matched remote brain tissue from the same patients was unavailable. All stroke and control samples were obtained from the middle temporal gyrus, and the stroke specimens represented peri‐infarct tissue, minimizing anatomical sampling differences between groups. However, the lack of paired peri‐infarct and remote tissues, together with the unavailable exact interval from stroke onset to death, limits the spatial and temporal interpretation of the human findings. Future studies using paired samples will be important for defining the distribution of β‐adducin loss in the human ischemic brain. Moreover, the translational implications of the FTO‐β‐adducin‐PLA2G4A pathway should also be interpreted cautiously. Direct manipulation of FTO or restoration of β‐adducin may be difficult to achieve therapeutically. By contrast, our post‐ischemic AACOCF3 experiments provide proof‐of‐concept that pharmacological inhibition of PLA2G4A, a more tractable downstream target, can attenuate the aggravated ischemic injury associated with β‐adducin deficiency. Further studies are required to define the therapeutic window, optimize dosing, assess safety, and determine whether PLA2G4A inhibition is effective in broader stroke settings. Finally, the current study primarily focused on the acute phase of ischemic injury, during which neuronal death and lysosomal dysfunction are most pronounced. Consequently, behavioral assessments were limited to the first 1–3 days after MCAO. Although modulation of this pathway improved acute neurological outcomes and reduced ischemic injury, whether these effects translate into sustained neurological recovery, cognitive improvement, or long‐term brain repair remains to be determined.

In conclusion, β‐adducin protects against ischemic neuronal injury by limiting PLA2G4A accumulation at lysosomes and preserving lysosomal integrity. Ischemia‐associated FTO loss contributes to increased m^6^A modification and destabilizes *Add2* mRNA, thereby reducing β‐adducin expression and promoting PLA2G4A‐associated lysosomal damage. Together, these findings define an FTO‐β‐adducin‐PLA2G4A axis in ischemic injury and support further evaluation of PLA2G4A inhibition as a potential downstream therapeutic strategy.

## 4 Materials and Methods

### 4.1 Experimental Animals

All animal procedures were approved by the Institutional Animal Care and Use Committee of Affiliated Drum Tower Hospital, Medical School of Nanjing University (approval no. 2023AE01007), and were performed in accordance with institutional guidelines for the care and use of laboratory animals. Mice were housed in a specific‐pathogen‐free (SPF) facility under a 12‐h light/dark cycle at a controlled temperature, with ad libitum access to standard chow and water.

*Syn1*‐Cre mice were purchased from GemPharmatech (Nanjing, China). *Fto* transgenic (*Fto*‐Tg) mice were a generous gift from Professor Zengqiang Yuan at the University of South China. Genotyping of *Syn1*‐Cre mice was performed via PCR using two primer pairs (forward primer 1 5'‐AGCAGAGGAGTCGCGTCGTG‐3', reverse primer 1 5'‐CGCAGCAGGGTGTTGTAGGC‐3', forward primer 2 5'‐AGTCTTTCCCTTGCCTCTGCT‐3' and reverse primer 2 5'‐GGGTCTTCCACCTTTCTTCAG‐3'), yielding products of 666 bp for the Cre‐positive allele and 825 bp for the wild‐type (WT) allele. Only the heterozygous *Syn1*‐Cre offspring were used for subsequent investigations. Genotyping of *Fto*‐Tg mice was conducted using the forward primer 5′‐GAGGGGAGGGATAAGTGAGG‐3′ and reverse primer 5′‐CATCTTTGGGGGTCAGGTAA‐3′. Sex‐matched WT littermates were employed as controls. All mice were on the C57BL/6J background.

### 4.2 Human Postmortem Brain Tissue

Human postmortem brain tissues from patients with ischemic stroke or non‐neurological diseases were provided by the Chinese Brain Bank Center (Table 1, approval no. 2021‐scuec‐034). All samples analyzed in this study were collected from the middle temporal gyrus. Stroke samples were obtained from peri‐infarct tissue of patients with massive cerebral infarction, whereas anatomically matched middle temporal gyrus tissues from donors without neurological disease were used as controls. The exact interval between stroke onset and death was unavailable. Written informed consent was obtained from all donors before donation. Unfixed brain tissues were processed as described in a previous study and stored at −80°C until subsequent immunostaining [62].

**TABLE 1:** Information on human postmortem brain samples.

Group	Age	Gender	Date of death	Cause of death	Brain region	Tissue location relative to infarct	Postmortem interval (PMI)	Stroke onset‐to‐death interval
Control 1	61	Male	20 Apr‐19	Diabetic nephropathy; type 2 diabetes	Middle temporal gyrus	N/A	7 h 35 min	N/A
Control 2	64	Male	26 Aug 19	Hypertension	Middle temporal gyrus	N/A	9 h 16 min	N/A
Control 3	60	Female	10 Sep 19	Hypertension; diabetes	Middle temporal gyrus	N/A	6 h 28 min	N/A
Stroke 1	60	Female	10 May 19	Massive cerebral infarction	Middle temporal gyrus	Peri‐infarct tissue	5 h 22 min	Not available
Stroke 2	55	Male	17 Apr 19	Massive cerebral infarction	Middle temporal gyrus	Peri‐infarct tissue	5 h 50 min	Not available
Stroke 3	67	Male	7 Jan 19	Massive cerebral infarction	Middle temporal gyrus	Peri‐infarct tissue	7 h 06 min	Not available

*Note*: All control and ischemic stroke tissues were obtained from the middle temporal gyrus. Stroke specimens were obtained from peri‐infarct regions. PMI was defined as the interval between the recorded time of death and the start of brain dissection. The exact interval between stroke onset and death was not available from the clinical records. N/A, not applicable.

### 4.3 Adeno‐Associated Virus (AAV) Injection

For conditional neuronal *Add2* knockdown, an shRNA targeting *Add2* (target sequence: 5'‐GCATGCCCATACGGATTGAAA‐3') was cloned into the pAAV‐CMV‐DIO‐mCherry‐mir30shRNA‐WPRE vector and packaged into the AAV‐PHP.eB virus by OBiO (Shanghai, China). Male *Syn1*‐Cre mice received a single intravenous tail‐vein injection of the packaged AAV‐PHP.eB virus at 6 weeks of age and were used for subsequent experiments at 9 weeks of age. Each mouse received 5 × 10^11^ viral genomes diluted in sterile PBS. Control mice were injected with an equivalent dose of the corresponding scrambled control AAV.

For neuronal *Add2* knockdown in *Fto*‐Tg mice, the same *Add2*‐targeting sequence (5'‐GCATGCCCATACGGATTGAAA‐3') was cloned into the pAAV‐hSyn‐mCherry‐miR30shRNA‐WPRE vector and packaged into AAV‐PHP.eB. Male *Fto*‐Tg mice received the virus by tail‐vein injection using the same dose, injection procedure, and experimental interval as described above (5 × 10^11^ viral genomes per mouse at 6 weeks of age, followed by a 3‐week expression period before subsequent experiments). The corresponding hSyn‐driven scrambled AAV was administered to control groups at the same dose.

### 4.4 Middle Cerebral Artery Occlusion (MCAO)

Transient focal cerebral ischemia was induced by intraluminal occlusion of the right middle cerebral artery (MCA) as previously described [63]. Briefly, eight‐ to nine‐week‐old mice were anesthetized with isoflurane. A monofilament suture was inserted into the right common carotid artery and carefully advanced to occlude the origin of the right MCA, which was confirmed by a precipitous decrease in regional cerebral blood flow. The occlusion was maintained for 1 h and then removed to allow for reperfusion. Core body temperature of the mice was maintained at 36.5°C–37.5°C throughout the surgery using a temperature‐controlled heating system.

### 4.5 AACOCF3 Administration

To pharmacologically inhibit PLA2G4A in vivo, AACOCF3 was administered to mice subjected to MCAO at a dose of 25 mg/kg by intraperitoneal injection [64, 65]. The first dose was given 3 h after MCAO, followed by additional injections on days 1 and 2. Control mice received the corresponding vehicle. PLA2G4A enzymatic activity was measured to confirm effective pharmacological inhibition.

### 4.6 PLA2G4A Enzymatic Activity Assay

PLA2G4A enzymatic activity was measured using a cPLA2 Assay Kit (Cayman Chemical, USA) according to the manufacturer's instructions. To minimize interference from calcium‐independent PLA2 (iPLA2), samples were preincubated with 5 µm bromoenol lactone before the assay. Briefly, equal amounts of protein were incubated with arachidonoyl Thio‐PC substrate for 60 min at room temperature. After DTNB/EGTA was added, absorbance was measured at 414 nm.

### 4.7 Neurological Assessments

To evaluate the neurological deficits following MCAO, a battery of behavioral tests was performed at 1 and 3 days after MCAO. All behavioral assessments were conducted by an investigator blinded to group allocation.

#### 4.7.1 Rotarod Test

Motor coordination was evaluated using a rotarod apparatus (RWD Life Science, China). Mice were trained for three consecutive days before surgery. Mice that failed to remain on the rod at 40 rpm for 5 min during pretraining were excluded from the study. During the test, the rod accelerated from 0 to 40 rpm over 5 min, and the latency to fall from the rotating rod was recorded in seconds.

#### 4.7.2 Foot‐Fault Test

To assess forelimb motor coordination, the foot fault test was employed. Mice were pre‐trained for 3 days by allowing them to habituate on an elevated stainless‐steel grid (12 × 12 mm^2^ apertures) for 5 min. During the test, mice were allowed to freely explore the grid for 3 min. A foot fault was recorded when the left forelimb slipped through the grid. The foot fault rate was calculated as the ratio of missed steps to the total number of steps taken with the left forelimb.

#### 4.7.3 Grip Strength Test

Forelimb grip strength was measured using a grip strength meter (GS3, Bioseb, France). Briefly, the mice were held by the tail and lowered toward the horizontally positioned apparatus. Once the mice firmly grasped the T‐bar with their forelimbs, they were smoothly pulled backward in the horizontal plane. Each mouse was tested 5 times, and the maximal peak tension was recorded in grams.

#### 4.7.4 Modified Neurological Severity Score (mNSS)

Global neurological deficits were graded using the mNSS, which evaluates motor, sensory, reflex, and balance functions. The mNSS was graded on a scale of 0 to 18, where a score of 0 indicates normal performance and a score of 18 represents the most severe neurological impairment.

### 4.8 Magnetic Resonance Imaging (MRI)

The brain infarct volume at 1 and 3 days post‐MCAO was determined using a 9.4T MRI scanner (BioSpec 94/20 USR, Bruker) at the Department of Radiology, Nanjing Drum Tower Hospital. Briefly, mice were anesthetized with 2.5%–3% isoflurane and subjected to *T*_2_‐weighted MRI with the following parameters: slice thickness = 0.7 mm (22 adjacent slices), TR/TE = 2500 ms/33 ms; matrix size = 256 × 256; and field of view [FOV] = 20 × 20 mm. The *T*_2_‐weighted images were processed using an MRI analysis calculator plugin of ImageJ software. The infarct volume was measured using ImageJ software and calculated as (contralateral hemisphere area − noninfarct ipsilateral hemisphere area)/(2 × contralateral hemisphere area) × 100%.

### 4.9 2,3,5‐Triphenyltetrazolium Chloride (TTC) Staining

Cerebral infarct volume was assessed by 2,3,5‐triphenyltetrazolium chloride (TTC, Sigma‐Aldrich) staining at 3 days after MCAO. Mice were euthanized, and the brains were rapidly removed and sectioned into 2‐mm‐thick coronal slices. Brain sections were incubated in 2% TTC in PBS at 37°C for 10 min, and the viable tissue was stained red. The infarct areas were quantified using ImageJ software and calculated as follows: (contralateral hemisphere area − noninfarct ipsilateral hemisphere area)/(2 × contralateral hemisphere area) × 100%.

### 4.10 Primary Cortical Neuron Culture

Primary cortical neurons were isolated from embryonic day 15–17 (E15–17) mouse embryos as previously described [66]. Briefly, cortices were dissected and digested with TrypLE. The dissociated cells were then seeded onto poly‐*D*‐lysine‐coated cell culture plates. The neurons were maintained in Neurobasal medium supplemented with B27 and GlutaMAX in a humidified incubator at 37°C with 5% CO_2_. Half of the culture medium was replaced every 2 days. The neurons were allowed to mature for 7 days in vitro before use.

### 4.11 Lentiviral Transduction

Lentiviral vectors encoding shRNAs targeting *Add2* (5'‐GCATGCCCATACGGATTGAAA‐3'), a second independent shRNA targeting *Add2* (sh*Add2*‐2, 5'‐CCTGATTAAAGTGAACATTCT‐3'), or *Pla2g4a* (5'‐GCACAGCTACATTCCCTGTAT‐3'), as well as vectors for *Add2* or wild‐type *Fto* overexpression, were synthesized by OBiO (Shanghai, China). A catalytically inactive FTO mutant (Mut‐FTO) carrying the H228A/D230A substitutions was also generated for lentiviral expression [67, 68]. Primary cortical neurons were transduced with the indicated lentiviruses at a multiplicity of infection of 10. After 12 h of infection, the virus‐containing medium was removed. The transduced neurons were then cultured for an additional 3 days prior to subsequent experiments.

### 4.12 Oxygen‐Glucose Deprivation and Reoxygenation (OGD/R)

To mimic ischemia‐reperfusion injury in vitro, primary cortical neurons were subjected to OGD/R. Briefly, neurons were incubated in glucose‐free Neurobasal A medium (Gibco, Thermo Fisher Scientific, USA) and transferred into a hypoxic chamber (flushed with 95% N_2_ and 5% CO_2_) at 37°C. Following a 30‐min OGD exposure, the medium was replaced with normal medium, and the neurons were returned to normoxic conditions for 6 or 12 h of reoxygenation.

### 4.13 BAPTA‐AM Treatment

To examine the effect of intracellular Ca^2^^+^ on the β‐adducin‐PLA2G4A association, primary cortical neurons were pretreated with the cell‐permeable Ca^2^^+^ chelator BAPTA‐AM (10 µm) for 30 min before OGD [69]. BAPTA‐AM was maintained throughout the 1‐h OGD period. At the end of OGD, neurons were collected and subjected to Co‐IP analysis.

### 4.14 Plasmid Construction and Cell Transfection

Full‐length *Add2*, *Pla2g4a*, *Dpp3*, and *Impact* cDNAs were amplified from a mouse cDNA library and inserted into the pcDNA3.1 expression vector using the ClonExpress II One Step Cloning Kit (Vazyme, Nanjing, China). C‐terminal 3 × FLAG‐tagged mouse *Add2*, as well as C‐terminal HA‐tagged *Pla2g4a*, *Dpp3*, and *Impact*, were constructed. HA‐tagged truncated *Pla2g4a* constructs encoding residues 1–123, 123–748, and 179–748 were synthesized by General Biol (Anhui, China). HEK293T cells were transiently co‐transfected with *Add2*‐Flag and the indicated HA‐tagged constructs using Lipofectamine 3000 (Invitrogen, USA) following the manufacturer's instructions.

### 4.15 Co‐Immunoprecipitation (Co‐IP)

Total protein from HEK293T cells, primary cortical neurons, or ischemic penumbral tissue collected from mice at 3 days after MCAO was extracted using IP lysis buffer (Beyotime Biotechnology, Beijing, China) supplemented with a protease inhibitor cocktail (MCE, USA). For exogenous Co‐IP in HEK293T cells, lysates were incubated with anti‐Flag or anti‐HA nanobody MagaroSe beads (AlpalifeBio, China). For endogenous Co‐IP in primary neurons and ischemic brain tissue, anti‐β‐adducin antibodies (Proteintech, #14640‐1‐AP) or control IgG were pre‐incubated with Protein A/G magnetic beads (MCE, USA) and then incubated with the lysates. After five washes with IP lysis buffer, bound proteins were eluted by boiling in 1× SDS loading buffer and analyzed by Western blot.

### 4.16 Immunoprecipitation‐Mass Spectrometry (IP‐MS)

For identification of β‐adducin‐associated proteins, Flag‐tagged β‐adducin protein complexes were enriched from primary neurons using anti‐Flag nanobody Magarose beads, with empty vector‐transduced neurons used as controls. Immunoprecipitated proteins were digested into peptides and subjected to LC‐MS/MS analysis by Novogene Co., Ltd. (Beijing, China). Peptides were analyzed by a Q Exactive HF‐X mass spectrometer with a Nanospray Flex ion source. Raw mass spectrometry data were processed by Proteome Discoverer 2.5 software for protein identification.

### 4.17 Immunofluorescence

All procedures were performed as previously described [70]. For brain sections, mice were deeply anesthetized and transcardially perfused with ice‐cold PBS followed by 4% paraformaldehyde (PFA). The brains were removed, post‐fixed in 4% PFA, and cryoprotected in graded sucrose solutions (10%, 20%, and 30%) in PBS. Coronal brain sections (20 µm in thickness) were prepared using a cryostat (Thermo Fisher Scientific, USA). For cultured neurons, primary neurons were washed with PBS and fixed with 4% PFA for 15 min at room temperature (*RT*). Both the brain sections and cells were incubated with blocking buffer (PBS containing 2% donkey serum, 0.3% Triton X‐100, and 0.1% BSA) for 1 h at RT. Subsequently, they were incubated overnight at 4°C with the following primary antibodies: anti‐β‐adducin (1: 200, Origene, #AP20545PU‐N), anti‐Iba1 (1: 500, Abcam, #ab5076), anti‐GFAP (1: 500, CST, #3670), anti‐Olig2 (1: 500, R&D Systems, #AF2418), anti‐S100β (1: 500, Abcam, #ab52642), anti‐PLA2G4A (1: 200, CST, #2832), anti‐LAMP1 (1: 400, CST, #99437), anti‐CTSB (1: 100, Santa Cruz, #sc‐365558) and anti‐CTSD (1: 100, Santa Cruz, #sc‐377299). Then, sections or cells were incubated with the corresponding fluorophore‐conjugated secondary antibodies at RT for 1 h in the dark. Cell nuclei were counterstained with DAPI (Beyotime Biotechnology, China). The FlexAble CoraLite antibody labeling kit (Proteintech) was utilized to generate a fluorophore‐conjugated primary antibody complex to reduce nonspecific background associated with same‐species immunofluorescence staining. Finally, images were captured using an Olympus FV3000 confocal laser scanning microscope (Olympus, Tokyo, Japan).

### 4.18 Fluoro‐Jade B (FJB) Staining

To evaluate neuronal degeneration following ischemic injury, FJB staining was performed on brain sections collected at 1 and 3 days post‐MCAO. Briefly, slides were dried at 50°C–60°C for 30 min and then sequentially immersed in 70% ethanol for 2 min and distilled water for 2 min. To suppress background fluorescence, the sections were incubated in 0.06% KMnO_4_ solution for 10 min. After incubation in a 0.001% FJB and 0.1% acetic acid solution for 15 min, the sections were washed with distilled water and thoroughly air‐dried. Finally, the slides were cleared in xylene and mounted using a neutral resin medium.

### 4.19 TUNEL Staining

Neuronal apoptosis was assessed using a TUNEL BrightGreen Apoptosis Detection Kit (Vazyme, Nanjing, China). Brain sections were collected at 1 and 3 days after MCAO. Sections were first subjected to immunofluorescence staining for NeuN to identify neurons. TUNEL labeling was then performed according to the manufacturer's instructions. Fluorescence images were acquired from the ischemic penumbra, and neuronal apoptosis was quantified as the percentage of TUNEL‐positive neurons among NeuN‐positive neurons.

### 4.20 Cell Viability Assay

To evaluate neuronal viability, the Cell Counting Kit‐8 (CCK‐8) assay (Fude Biological Technology, China) was utilized. Primary cortical neurons were seeded into 96‐well culture plates. Following lentiviral transduction and subsequent exposure to the OGD/R challenge (12 h of reoxygenation), cell viability was assessed according to the manufacturer's instructions. Briefly, after a 4‐h incubation with the CCK‐8 reagent, the absorbance was measured at 450 nm using a microplate reader (Tecan).

### 4.21 Lactate Dehydrogenase (LDH) Release Assays

Cellular injury was assessed by measuring LDH release into the culture medium using the Cytotoxicity Detection KitPLUS (LDH) (Roche Diagnostics, Germany) according to the manufacturer's instructions. Briefly, culture supernatants were collected after the indicated treatments and incubated with the reaction mixture provided in the kit. Absorbance was measured at 490 nm.

### 4.22 Calcein‐AM/PI Double Staining

Viability assessment was performed using the Calcein‐AM/PI double staining kit (Dojindo, Japan). Briefly, after 12 h of reoxygenation, neurons were incubated in PBS containing 2 µm Calcein‐AM and 4.5 µm PI for 15 min at 37°C in the dark. Images were immediately captured using a fluorescence microscope. Calcein‐AM‐positive viable neurons exhibited green fluorescence, whereas PI‐positive dead or dying neurons exhibited red fluorescence.

### 4.23 Annexin V‐FITC/7‐AAD Flow Cytometric Analysis

Cell apoptosis was assessed using an Annexin V‐FITC/7‐AAD Apoptosis Detection Kit (Vazyme, Nanjing, China) according to the manufacturer's instructions. Briefly, HT‐22 cells were collected, washed with PBS, and resuspended in binding buffer. The cells were then stained with Annexin V‐FITC and 7‐AAD and analyzed by flow cytometry. Annexin V‐FITC^+^/7‐AAD^−^ cells were considered early apoptotic, whereas Annexin V‐FITC^+^/7‐AAD^+^ cells were considered late apoptotic or membrane‐compromised cells. The percentage of Annexin V‐positive cells was used to quantify apoptosis.

### 4.24 Intracellular Calcium Imaging

To detect intracellular calcium overload during the OGD challenge, the fluorescent calcium indicator Fluo‐4 AM (Thermo Fisher Scientific, USA) was used. Briefly, primary cortical neurons were incubated with Neurobasal medium containing 4 µm Fluo‐4 AM at 37°C for 30 min and then washed. Baseline fluorescence was recorded in extracellular solution containing (in mm): 140 NaCl, 5 KCl, 1 CaCl_2_, 1 MgCl_2_, 10 HEPES, and 10 *D*‐glucose (pH adjusted to 7.4 with NaOH). The extracellular solution was replaced with OGD solution using a peristaltic pump (BT100‐2J, Longer Precision Pump Co., Ltd., Hebei, China). The OGD solution was formulated identically to the extracellular solution, except that *D*‐glucose was omitted and 1 mm sodium dithionite was added as an oxygen scavenger. Furthermore, the OGD solution was pre‐bubbled with 95% N_2_/5% CO_2_ for more than 15 min. The cytoplasmic calcium fluorescence was recorded at 0.2 Hz using a Hamamatsu digital imaging camera (Hamamatsu, C11440‐22U) at *RT*. Changes in fluorescence were normalized as *∆F*/*F_0_* = (*F_t_ *− *F_0_*)/*F_0_*, where *F_0_* is the baseline fluorescence, and *F_t_* is the fluorescence at time *t*.

### 4.25 Real‐Time Quantitative Polymerase Chain Reaction (qPCR)

Total RNA was extracted using the AG RNAex Pro Reagent (Accurate Biology, China) and reverse‐transcribed using Evo M‐MLV RT Mix Kit with gDNA Clean for qPCR Ver.2 (Accurate Biology, China) following the manufacturer's instructions. Subsequently, quantitative real‐time PCR was performed using the ChamQ Blue Universal SYBR qPCR Master Mix (Vazyme Biotech, China). The mRNA expression was calculated using the 2^−ΔΔ^ *^Ct^* method, with β‐actin serving as a housekeeping gene for normalization. Primer sequences are listed in Table 2.

**TABLE 2:** Primer sequences.

Primer		Sequence (5' to 3')
*Add2*	F	CTGACACTGATGGCGACAG
	R	AACGAGGGGGTTCGGAATTTC
*Add1*	F	CCAGGGACGACAGTGATGC
	R	AGGGGAGGCTACCTCTTCAG
*Add3*	F	AGCAGAGGAAGCGAGTCACT
	R	GCTTGGGTTGTGGCCTTTCT
*Fto*	F	TTCATGCTGGATGACCTCAATG
	R	GCCAACTGACAGCGTTCTAAG
*Pla2g4a*	F	CAGCACATTATAGTGGAACACCA
	R	AGTGTCCAGCATATCGCCAAA
*Alkbh5*	F	CGCGGTCATCAACGACTACC
	R	ATGGGCTTGAACTGGAACTTG
*Mettl3*	F	CTGGGCACTTGGATTTAAGGAA
	R	TGAGAGGTGGTGTAGCAACTT
*Mettl14*	F	CTGAGAGTGCGGATAGCATTG
	R	GAGCAGATGTATCATAGGAAGCC
*Wtap*	F	GAACCTCTTCCTAAAAAGGTCCG
	R	TTAACTCATCCCGTGCCATAAC

### 4.26 Western Blotting

Western blotting was performed as previously described [71]. Briefly, the collected cells or tissues were lysed in RIPA buffer supplemented with a protease and phosphatase inhibitor cocktail (MCE, USA). Protein concentrations were determined using a BCA Protein Assay Kit (Cwbio, China). Equal amounts of protein samples were separated by sodium dodecyl sulfate‐polyacrylamide gel electrophoresis (SDS‐PAGE), and subsequently transferred onto PVDF membranes (Millipore, Billerica, MA, USA). The membranes were blocked with 5% skim milk and then incubated overnight at 4°C with the specific primary antibodies. Following incubation with the HRP‐conjugated secondary antibodies, the protein bands were visualized with enhanced chemiluminescence (ECL). The primary antibodies used were anti‐β‐adducin (Proteintech, #14640‐1‐AP), α‐adducin (Santa Cruz, #sc‐33633), β‐actin (Bioworld, #AP0060), Caspase‐3 (Proteintech, #19677‐1‐AP), Caspase‐9 (CST, #9504), HA (CST, #3724), Flag (ABclonal, #AE005), PLA2G4A (CST, #2832), p‐PLA2G4A (Bioworld, #BS4052), LAMP1 (Abcam, #ab24170), GAPDH (Bioworld, #BS00243), CTSB (Proteintech, #12216‐1‐AP), CTSD (ABclonal, #A19680) and FTO (Proteintech, #27226‐1‐AP). The original blot images corresponding to Figures 1, 2, 3, 4, 5, 6, 7 are provided in Figures S15–S17, and those corresponding to Figures S2–S14 are provided in Figures S18 and s19.

### 4.27 EGFP‐LGALS3 Puncta Assay

Lysosomal membrane damage was assessed using an EGFP‐linker‐LGALS3 puncta reporter assay. Primary cortical neurons were first transfected with the EGFP‐linker‐LGALS3 construct. Two days later, cells were transduced with the indicated lentiviruses and subsequently subjected to the indicated treatments. Cells were then fixed and immunostained for LAMP1 to visualize lysosomes. Fluorescence images were acquired using an Olympus FV3000 confocal microscopy (Olympus, Tokyo, Japan), and LAMP1‐positive EGFP‐LGALS3 puncta were quantified on a per‐cell basis.

### 4.28 Subcellular Fractionation and Lysosome Enrichment

For subcellular fractionation, primary neurons or ischemic penumbral tissues collected at 3 days after MCAO were homogenized in buffer containing 0.32 m sucrose and 10 mm HEPES supplemented with a protease and phosphatase inhibitor cocktail. The homogenates were centrifuged at 800 × *g* for 10 min at 4°C to clear cell debris and nuclei. The resulting supernatant was subsequently centrifuged at 20 000 × *g* for 20 min at 4°C. The supernatant was further ultracentrifuged at 100 000 × *g* for 1 h at 4°C to obtain the cytosolic fraction.

For the isolation of purified lysosomes, the Lysosome Enrichment Kit (Thermo Fisher Scientific, USA) was employed. According to the manufacturer's instructions, the prepared sample extract was subjected to discontinuous density gradient centrifugation. The distinct lysosomal band was extracted to obtain a highly purified lysosomal fraction. Both the isolated cytosolic and lysosomal fractions were subsequently subjected to Western blot analysis or a CTSB activity assay.

### 4.29 CTSB Activity Assay

To assess CTSB enzymatic activity within the cytosolic and lysosomal fractions, the CTSB Activity Assay Kit (Fluorometric) (Abcam, UK) was employed. Briefly, equal amounts of protein were incubated with the CTSB reaction buffer and substrate Ac‐RR‐AFC at 37°C for 1 h in the dark. The fluorescence intensity was measured using a fluorescence microplate reader at *E*_x_/*E*_m_ = 400/505 nm.

### 4.30 Assessment of Lysosomal Acidification

LysoSensor Green DND‐189 and LysoTracker Red DND‐99 (Thermo Fisher Scientific, USA) probes were used to visualize and evaluate lysosomal function. After OGD/R insult, primary cortical neurons were incubated with 1 µm LysoSensor and 1 µm LysoTracker in Neurobasal medium at 37°C for 30 min in the dark. The nuclei were counterstained with Hoechst 33342 (Beyotime Biotechnology, Shanghai, China). The live neurons were imaged using an Olympus FV3000 microscope.

### 4.31 Dot Blot Assay

To evaluate global RNA m^6^A levels, a dot blot assay was performed as previously described [62]. Briefly, equal amounts of RNA were denatured at 95°C for 3 min and immediately chilled on ice. The denatured RNA samples were spotted onto nylon membranes. After UV crosslinking, the membranes were stained with 0.02% methylene blue and imaged. The membranes were then blocked with 5% skim milk and then incubated with anti‐m^6^A antibody (Proteintech, #68055‐1‐Ig) overnight at 4°C. After incubation with HRP‐conjugated secondary antibody, the m^6^A signals were visualized with ECL.

### 4.32 MeRIP‐qPCR

MeRIP‐qPCR assay was conducted to quantify the m^6^A modification on *Add2* mRNA. Briefly, cells were lysed in IP lysis buffer supplemented with RNase inhibitor (0.4 U/µL). Following RNA fragmentation, 10% of the fragmented RNA lysate was reserved as the input control. The remaining lysate was then incubated with magnetic beads pre‐conjugated with either anti‐m^6^A antibody or mouse IgG. After thorough washing steps, the immunoprecipitated RNA complexes along with the reserved input samples were subjected to RNA extraction using the TRIzol reagent. Finally, the enrichment of m^6^A was analyzed via qPCR.

### 4.33 RNA Stability Assay

Primary cortical neurons were treated with 5 µg/mL actinomycin D (MCE, USA) to inhibit de novo RNA transcription. Total RNA was extracted at the indicated time points (0, 2, 6, and 12 h) using the AG RNAex Pro Reagent and prepared for qPCR analysis. The relative remaining RNA levels at each time point were normalized to the RNA level at 0 h. The transcript half‐life was calculated using a one‐phase exponential decay model.

### 4.34 Statistical Analysis

All quantitative data are expressed as the mean ± standard error of the mean (SEM). Statistical analyses were performed using GraphPad Prism software. One‐way or two‐way analysis of variance (ANOVA) followed by Sidak's multiple comparisons test was employed to determine differences among groups, while the unpaired two‐tailed Student's *t*‐test was used for two‐group comparisons. A value of *P* < 0.05 was considered statistically significant.

## Author Contributions

X.C. and Y.X. conceived, designed, and directed the project. H‐q.L., P‐y.L., D.W., F‐y.M., Y.B., S‐n.X., H‐y.Y. and X‐y.B. performed the experiments and analyzed the data. X.C. and H‐q.L. wrote the manuscript. Y.X. and P‐y.L. revised the manuscript.

## Funding

This research was supported by the Natural Science Foundation of Jiangsu Province BK20240118 (to X.C), the National Natural Science Foundation of China 82371326 (to X.C), U25A2064 (to Y.X) and 82130036 (to Y.X), the STI2030‐Major Projects 2022ZD0211800 (to Y.X), Jiangsu Province Key Medical Discipline ZDXK202216 (to Y.X).

## Conflicts of Interest

The authors declare no conflicts of interest.

## Supporting information




**Supporting File**: advs78183‐sup‐0001‐SuppMat.docx.

## Data Availability

The data that support the findings of this study are available from the corresponding author upon reasonable request.
