# Supplementary material for: β‐Adducin Restrains PLA2G4A‐Dependent Lysosomal Membrane Permeabilization and Neuronal Death in Ischemic Stroke
Source: Adv Sci (Weinh). 2026 Oct 6:e78183. Online ahead of print. doi: 10.1002/advs.78183 (PMC13643116; doi:10.1002/advs.78183)
Supplement: Supplementary file 1 [file ADVS-9999-e78183-s001.docx]

**Supplementary Materials for**

**β-adducin restrains PLA2G4A-dependent lysosomal membrane permeabilization and neuronal death in ischemic stroke**

**This file includes:**

**Figure S1 to S19**

Figure S1. Widespread expression of *Add1* in the brain and the downregulation of β-adducin in neurons following ischemic stroke.

Figure S2. Validation of the neuron-specific *Add2* knockdown *in vivo* model.

Figure S3. The expression of adducin in OGD/R-treated primary neurons and HT-22 cells.

Figure S4. Validation of *Add2* genetic manipulation efficiency and representative intracellular calcium imaging in primary neurons.

Figure S5. *Add2* knockdown exacerbates ischemic and excitotoxic injury in the HT-22 neuronal cell line.

Figure S6. *Add2* overexpression alleviates ischemic and excitotoxic injury in the HT-22 neuronal cell line.

Figure S7. β-adducin associates with PLA2G4A in ischemic brain and under altered intracellular Ca²⁺ conditions.

Figure S8. β-adducin limits lysosome-associated PLA2G4A activity by regulating its subcellular distribution.

Figure S9. β-adducin deficiency promotes PLA2G4A-associated lysosomal dysfunction and cathepsin D redistribution.

Figure S10. Validation of *Add2* loss-of-function phenotypes using a second independent shRNA.

Figure S11. Effects of β-adducin deficiency on CTSD subcellular distribution and AACOCF3 on PLA2G4A activity after MCAO.

Figure S12. Expression of m6A regulators after MCAO and validation of neuronal *Add2* knockdown in *Fto*-Tg mice.

Figure S13. Expression profile of m6A RNA methylation regulators and validation of genetic interventions in primary neurons.

Figure S14. FTO overexpression reduces PLA2G4A lysosomal accumulation and preserves lysosomal integrity after OGD/R.

Figure S15. Original blots related to Figure 1-3.

Figure S16. Original blots related to Figure 4.

Figure S17. Original blots related to Figure 5-7.

Figure S18. Original blots related to Figure S2-S9.

Figure S19. Original blots related to Figure S10-S14.

**Additional Figures:**

**Figure S1. Widespread expression of *Add1* in the brain and the downregulation of β-adducin in neurons following ischemic stroke.**

**A** UMAP plots from Reference ^[31]^ illustrating the distribution of *Add1* expression (encoding α-adducin) across the identified cell clusters. **B** Immunostaining of β-adducin (green) and NeuN (red) to evaluate the protein expression of β-adducin in neurons at 1 and 3 days after MCAO (n = 3, one-way ANOVA, ***p* < 0.01, ****p* < 0.001). Scale bar, 50 µm. Data are expressed as the mean ± SEM.

**Figure S2. Validation of the neuron-specific *Add2* knockdown *in vivo* model.**

**A** Genotyping PCR analysis identifying *Syn1*-Cre mice. **B** Brain sections from mice injected with AAV-sh*Add2* were examined for AAV-derived mCherry fluorescence (red) and co-stained with cell-type-specific markers (green), including Iba-1 (microglia), S100β (astrocytes), and Olig2 (oligodendrocytes). Scale bar, 50 µm. **C** qPCR analysis confirming *Add2* mRNA knockdown following AAV-sh*Add2* injection (n = 4, unpaired *t* test, ***p* < 0.01). **D** Western blot analysis confirming β-adducin protein knockdown following AAV-sh*Add2* injection (n = 4, unpaired *t* test, **p* < 0.05). Data are expressed as the mean ± SEM. Original blot can be found in Figure S18.

**Figure S3. The expression of adducin in OGD/R-treated primary neurons and HT-22 cells.**

**A** The protein expression of α-adducin in primary neurons following OGD/R treatment (n = 3, unpaired *t* test, ns, not significant). **B** The protein expression of β-adducin and α-adducin in the HT-22 murine hippocampal neuronal cell line exposed to OGD/R insult (n = 3, unpaired *t* test, **p* < 0.05, ns, not significant). Data are expressed as the mean ± SEM. Original blot can be found in Figure S18.

**Figure S4. Validation of *Add2* genetic manipulation efficiency and representative intracellular calcium imaging in primary neurons.**

**A, B** qPCR (A) and western blot analysis (B) confirming the efficient knockdown of *Add2* mRNA and β-adducin protein, respectively, following transfection with sh*Add2* in primary neurons (n = 3, unpaired *t* test, **p* < 0.05, ***p* < 0.01). **C, D** qPCR (C) and western blot analysis (D) validating the successful overexpression of *Add2* mRNA and β-adducin protein respectively following transfection with oe*Add2* in primary neurons (n = 3, unpaired *t* test, ***p* < 0.01, ****p* < 0.001). **E, F** LDH release assay assessing cytotoxicity in primary cortical neurons following *Add2* knockdown (E) or overexpression (F) under normoxic and OGD/R conditions. (n = 6, one-way ANOVA, ****p* < 0.001). **G,** **H** Pseudocolor images of intracellular calcium levels at 0 min and 60 min of OGD in primary neurons with *Add2* knockdown (G) or overexpression (H). Data are expressed as the mean ± SEM. Original blot can be found in Figure S18.

**Figure S5. *Add2* knockdown exacerbates ischemic and excitotoxic injury in the HT-22 neuronal cell line.**

**A, B** qPCR (A) and western blot analysis (B) evaluating the knockdown efficiency of three distinct shRNAs targeting *Add2* and the reduction of β-adducin protein by the third sh*Add2* sequence in HT-22 cells (n = 3, one-way ANOVA for qPCR, unpaired *t* test for western blot analysis, **p* < 0.05, ****p* < 0.001). **C** Cell viability assays assessing the susceptibility of control and *Add2* knockdown HT-22 cells to OGD/R or 5 mM glutamate toxicity (n = 6, one-way ANOVA, ****p* < 0.001, ns, not significant). **D** Annexin V and 7-AAD double staining assessing cellular apoptosis across the specified treatment groups (n = 3, one-way ANOVA, ***p* < 0.01, ****p* < 0.001, ns, not significant). **E** The mean fluorescence intensity (MFI) of Fluo4-AM evaluating intracellular calcium overload in HT-22 cells subjected to OGD/R or glutamate exposure (n = 3, one-way ANOVA, ***p* < 0.01, ns, not significant). **F** The protein expression of cleaved Caspase-3, Caspase-3, cleaved Caspase-9 and Caspase-9 in HT-22 cells following OGD/R stress (n = 3, one-way ANOVA, **p* < 0.05, ****p* < 0.001, ns, not significant). Data are expressed as the mean ± SEM. Original blot can be found in Figure S18.

**Figure S6. *Add2* overexpression alleviates ischemic and excitotoxic injury in the HT-22 neuronal cell line.**

**A, B** qPCR (A) and western blot analysis (B) confirming successful overexpression of *Add2* mRNA and β-adducin protein, respectively, following transfection with oe*Add2* in HT-22 cells (n = 3, unpaired *t* test, **p* < 0.05, ****p* < 0.001). **C** Cell viability assays evaluating the protective effects of *Add2* overexpression against OGD/R or 5 mM glutamate toxicity in HT-22 cells (n = 3, one-way ANOVA, ****p* < 0.001, ns, not significant). **D** Annexin V and 7-AAD double staining assessing cellular apoptosis across the specified treatment groups (n = 3, one-way ANOVA, ****p* < 0.001, ns, not significant). **E** The MFI of Fluo4-AM evaluating the suppression of intracellular calcium overload by oe*Add2* in HT-22 cells subjected to OGD/R or glutamate exposure (n = 3, one-way ANOVA, **p* < 0.05, ns, not significant). **F** The protein expression of cleaved Caspase-3, Caspase-3, cleaved Caspase-9 and Caspase-9 in HT-22 cells following OGD/R stress (n = 3, one-way ANOVA, ****p* < 0.001, ns, not significant). Data are expressed as the mean ± SEM. Original blot can be found in Figure S18.

**Figure S7. β-adducin associates with PLA2G4A in ischemic brain and under altered intracellular Ca²⁺ conditions.**

**A** Representative immunofluorescence images illustrating the colocalization of β-adducin (green) and PLA2G4A (red) in primary neurons. Scale bar, 20 μm. **B** Co-immunoprecipitation analysis of endogenous β-adducin and PLA2G4A in cortical tissue collected from mice at 3 days after MCAO. Brain lysates were immunoprecipitated with an anti-β-adducin antibody or control IgG, followed by immunoblotting for PLA2G4A and β-adducin. Input lysates were included as controls. **C** Co-immunoprecipitation analysis of phosphorylated PLA2G4A (p-PLA2G4A) associated with β-adducin in primary cortical neurons. Cell lysates were immunoprecipitated with an anti-β-adducin antibody or control IgG, followed by immunoblotting for p-PLA2G4A and β-adducin. Input lysates were included as controls. **D** Co-immunoprecipitation analysis of the β-adducin-PLA2G4A association in primary cortical neurons subjected to OGD in the presence or absence of BAPTA-AM (10 μM). Cell lysates were immunoprecipitated with an anti-β-adducin antibody or control IgG, followed by immunoblotting for PLA2G4A and β-adducin. Input lysates were included as controls. Original blot can be found in Figure S18.

**Figure S8.** **β-adducin limits lysosome-associated PLA2G4A activity by regulating its** **subcellular distribution.**

**A, B** Representative western blots and quantitative analyses of total PLA2G4A and phosphorylated PLA2G4A in primary cortical neurons following *Add2* knockdown (A) or *Add2* overexpression (B) under basal and OGD/R conditions. Total PLA2G4A was normalized to β-actin, and the relative phosphorylation level was expressed as the p-PLA2G4A/PLA2G4A ratio (n = 3, one-way ANOVA, ns, not significant). **C, D** PLA2G4A enzymatic activity measured separately in the cytosolic and lysosomal fractions of primary cortical neurons following *Add2* knockdown (C) or *Add2* overexpression (D) under basal and OGD/R conditions (n = 3, one-way ANOVA, ****p* < 0.001, ns, not significant). Data are expressed as the mean ± SEM. Original blot can be found in Figure S18.

**Figure S9. β-adducin deficiency promotes PLA2G4A-associated lysosomal dysfunction and cathepsin D redistribution.**

**A, B** qPCR (A) and western blot analysis (B) confirming the simultaneous knockdown efficiency of *Add2* and *Pla2g4a* at both the mRNA and protein levels in primary neurons co-transfected with sh*Add2* and sh*Pla2g4a* (n = 3, unpaired *t* test, **p* < 0.05, ***p* < 0.01, ****p* < 0.001). **C, D** Immunofluorescence images (C) and quantification (D) of the colocalization between CTSD (green) and LAMP1 (red) in primary neurons (n = 3, one-way ANOVA, **p* < 0.05, ***p* < 0.01, ns, not significant). Scale bar, 20 µm. **E, F** Fluorescence imaging (E) using LysoSensor (green) and LysoTracker (red), together with their respective quantitative analyses (F) to evaluate lysosomal acidification and LysoTracker fluorescence intensity following the specified interventions and OGD/R exposure (n = 3, one-way ANOVA, ***p* < 0.01, ****p* < 0.001, ns, not significant). Scale bar, 20 µm. Data are expressed as the mean ± SEM. Original blot can be found in Figure S18.

**Figure S10. Validation of *Add2* loss-of-function phenotypes using a second independent shRNA.**

**A** qPCR analysis of *Add2* mRNA expression in primary cortical neurons transduced with NC or a second independent *Add2*-targeting shRNA (sh*Add2*-2; 5’-CCTGATTAAAGTGAACATTCT-3’) (n = 3, unpaired *t* test, ***p* < 0.01). **B** Representative western blots and quantitative analysis of β-adducin protein expression following transduction with NC or sh*Add2*-2 (n = 3, unpaired *t* test, ****p* < 0.001). **C** Representative immunofluorescence images of PLA2G4A and LAMP1 in NC- and sh*Add2*-2-transduced primary cortical neurons under basal and OGD/R conditions. PLA2G4A is shown in green, LAMP1 in red, and nuclei in blue. Scale bar, 20 μm. **D** Representative fluorescence images of EGFP-LGALS3 and LAMP1 in NC- and sh*Add2*-2-transduced primary cortical neurons under basal and OGD/R conditions. EGFP-LGALS3 is shown in green, LAMP1 in red, and nuclei in blue. Scale bar, 20 μm. **E** Quantification of EGFP-LGALS3 puncta per cell under the indicated conditions (n = 3, one-way ANOVA, **p* < 0.05). **F, G** CTSB activity measured in the cytosolic (F) and lysosomal (G) fractions of NC- and sh*Add2*-2-transduced primary cortical neurons under basal and OGD/R conditions (n = 3, one-way ANOVA, **p* < 0.05, ***p* < 0.01). **H** Representative Calcein-AM/PI staining images and quantification of PI-positive cells in NC- and sh*Add2*-2-transduced primary cortical neurons under basal and OGD/R conditions. Scale bar, 100 μm (n = 3, one-way ANOVA, ****p* < 0.001). Data are expressed as the mean ± SEM. Original blot can be found in Figure S19.

**Figure S11. Effects of β-adducin deficiency on CTSD subcellular distribution and AACOCF3 on PLA2G4A activity after MCAO.**

**A, B** Subcellular fractionation analysis displaying the distribution of Cathepsin D (CTSD) in the cytosolic and lysosomal fractions isolated from the ischemic penumbra of sham-operated mice and MCAO mice receiving AAV-NC or AAV-sh*Add2* (n = 4, one-way ANOVA, **p* < 0.05 versus sham; ^##^*p* < 0.01 versus MCAO + AAV-NC). GAPDH and LAMP1 were used as markers for the cytosolic and lysosomal fractions, respectively. **C** PLA2G4A enzymatic activity in ischemic brain tissues from the indicated groups (n = 6, one-way ANOVA, ns, not significant, ^#^*p* < 0.05 versus AAV-sh*Add2* + vehicle). Data are expressed as the mean ± SEM. Original blot can be found in Figure S19.

**Figure S12. Expression of m6A regulators after MCAO and validation of neuronal *Add2* knockdown in *Fto*-Tg mice.**

**A** Relative mRNA expression levels of classical m6A demethylases (*Fto*, *Alkbh5*) and methyltransferases (*Mettl3*, *Mettl14*, *Wtap*) after ischemic injury (n = 6, one-way ANOVA, **p* < 0.05, ***p* < 0.01, ****p* < 0.001, ns, not significant). **B** Representative genotyping PCR analysis identifying *Fto*-Tg mice. **C, D** qPCR (C) and western blot analyses (D) confirming the overexpression of FTO at both the mRNA and protein levels in the brain tissues of *Fto*-Tg mice compared to WT littermates (n = 4, unpaired *t* test, ***p* < 0.01, ****p* < 0.001). **E** Representative immunofluorescence images showing mCherry expression from AAV-sh*Add2* together with NeuN, Iba1, S100β, or Olig2 in mouse brain sections. Nuclei were counterstained with DAPI. Scale bar, 50 μm. **F** qPCR analysis of *Add2* and *Fto* mRNA expression in WT + AAV-NC, *Fto*-Tg + AAV-NC, and *Fto*-Tg + AAV-sh*Add2* mice (n = 6, one-way ANOVA, ns, not significant, ****p* < 0.001 versus WT + AAV-NC; ^###^*p* < 0.001 versus *Fto*-Tg + AAV-NC). **G** Representative western blots and quantitative analyses of β-adducin and FTO protein expression in the indicated groups (n = 6, one-way ANOVA, ns, not significant, ****p* < 0.001 versus WT + AAV-NC; ^##^*p* < 0.01 versus *Fto*-Tg + AAV-NC). Data are expressed as the mean ± SEM. Original blot can be found in Figure S19.

**Figure S13. Expression profile of m6A RNA methylation regulators and validation of genetic interventions in primary neurons.**

**A** Dot blot analysis of global m6A RNA methylation levels in primary neurons subjected to OGD/R for indicated time points (3, 6, 12, and 24 h) compared to control. **B** qPCR analysis of the mRNA expression levels of classical m6A demethylases (*Fto*, *Alkbh5*) and methyltransferases (*Mettl3*, *Mettl14*, *Wtap*) in primary neurons following OGD/R injury (n = 4, one-way ANOVA, **p* < 0.05, ***p* < 0.01, ****p* < 0.001, ns, not significant). **C, D** qPCR (C) and western blot (D) analyses of *Fto* mRNA and FTO protein expression, respectively, in primary neurons following *Fto* overexpression (n = 3, unpaired *t* test, ****p* < 0.001). **E-G** qPCR (E) and western blot analyses (F, G) of *Add2*/β-adducin and *Fto*/FTO expression in primary neurons following combined *Add2* knockdown and *Fto* overexpression (n = 3, unpaired *t* test, **p* < 0.05, ***p* < 0.01, ****p* < 0.001). **H** Dot blot analysis of global m6A levels in neurons expressing NC, WT-FTO, or Mut-FTO, with methylene blue staining used as a loading control. Data are expressed as the mean ± SEM. Original blot can be found in Figure S19.

**Figure S14. FTO overexpression reduces PLA2G4A lysosomal accumulation and preserves lysosomal integrity after OGD/R.**

**A** qPCR analysis of *Pla2g4a* mRNA expression in primary cortical neurons transduced with NC or oe*Fto* vectors under basal and OGD/R conditions (n = 3 for NC and oe*Fto* under basal conditions; n = 4 for NC and oe*Fto* under OGD/R conditions, one-way ANOVA, ns, not significant). **B** Representative western blots and quantitative analysis of total PLA2G4A protein expression in primary cortical neurons under the indicated conditions. β-actin was used as the loading control (n = 3, one-way ANOVA, ns, not significant). **C** Subcellular fractionation and western blot analysis showing the distribution of PLA2G4A in the cytosolic and lysosomal fractions of primary cortical neurons under the indicated conditions. GAPDH and LAMP1 were used as cytosolic and lysosomal fraction markers, respectively (n = 3, one-way ANOVA, ns, not significant; **p* < 0.05). **D** Representative immunofluorescence images of PLA2G4A and LAMP1 in primary cortical neurons transduced with NC or oe*Fto* vectors under basal and OGD/R conditions. PLA2G4A is shown in green, LAMP1 in red, and nuclei in blue. Scale bar, 20 μm. **E** Representative fluorescence images and quantification of EGFP-LGALS3 puncta in primary cortical neurons co-stained with LAMP1 under basal and OGD/R conditions (n = 3, one-way ANOVA, ***p* < 0.01). EGFP-LGALS3 is shown in green, LAMP1 in red, and nuclei in blue. LGALS3 puncta per cell were quantified. Scale bar, 20 μm. **F** Subcellular fractionation and western blot analysis of CTSB and CTSD distribution in the cytosolic and lysosomal fractions under the indicated conditions (n = 3, one-way ANOVA, **p* < 0.05). GAPDH and LAMP1 were used as cytosolic and lysosomal fraction markers, respectively. **G** Representative immunofluorescence images of CTSD and LAMP1 and quantitative analysis of CTSD/LAMP1 colocalization in primary cortical neurons under basal and OGD/R conditions (n = 3, one-way ANOVA, ****p* < 0.001). CTSD is shown in green, LAMP1 in red, and nuclei in blue. Scale bar, 20 μm. Data are expressed as the mean ± SEM. Original blot can be found in Figure S19.

**Figure S15. Original blots related to Figure 1-3.**

**Figure S16. Original blots related to Figure 4.**

**Figure S17. Original blots related to Figure 5-7.**

**Figure S18. Original blots related to Figure S2-S9.**

**Figure S19. Original blots related to Figure S10-S14.**

**Reference**

31. M. Ximerakis, S. L. Lipnick, B. T. Innes, et al., “Single-Cell Transcriptomic Profiling of the Aging Mouse Brain,” *Nature Neuroscience* *22*, no. 10 (2019): 1696–1708, https://doi.org/10.1038/s41593-019-0491-3.
